# The topology of molecular representations and its influence on machine learning performance

**DOI:** 10.1186/s13321-025-01045-w

**Published:** 2025-07-21

**Authors:** Florian Rottach, Sebastian Schieferdecker, Carsten Eickhoff

**Affiliations:** 1 Central Data Science, Boehringer Ingelheim GmbH, Biberach/Riss, Germany; 2 Computational Toxicology, Boehringer Ingelheim Pharma GmbH & Co. KG, Biberach/Riss, Germany; 3 School of Medicine, University of Tübingen, Tübingen, Germany

**Keywords:** Molecular property prediction, QSAR, Persistent homology, Topological data analysis, Generalizability

## Abstract

Advancements in cheminformatics have led to numerous methods for encoding molecules numerically. The choice of molecular representation impacts the accuracy and generalizability of learning algorithms applied to chemical datasets. Designing and selecting the appropriate representation often lacks a systematic approach and follows computationally exhaustive empirical testing. Moreover, research has shown that deep learning models do not substantially outperform traditional approaches across many tasks with no clear explanation for this shortfall. In this work, we present TopoLearn, a model that predicts the effectiveness of representations on datasets based on the topological characteristics of the corresponding feature space. Using interpretability techniques, we find that persistent homology descriptors are linked with the error metrics of trained machine learning models, offering a new method to better understand and select molecular representations.

**Scientific contribution** Our research is the first to establish an empirical connection between the topology of feature spaces and the machine learning performance of molecular representations. In addition, we facilitate future research endeavors by providing open access to our developed model.

**Supplementary Information:**

The online version contains supplementary material available at 10.1186/s13321-025-01045-w.

## Introduction

The development of novel medicine is a complex, costly, and uncertain process that presents many challenges for the pharmaceutical industry. Developing a new drug can take up to 15 years and the costs can rise to several billion US dollars [1, 2]. In addition, most drug candidates fail in the later phases of clinical trials and drug approval [3, 4], which is mostly attributed to insufficient drug-like properties, unacceptable toxicity and lack of clinical efficacy [5]. Traditional in vivo or in vitro experiments for measuring drug properties are time-consuming and expensive; therefore computer-aided methods have become ubiquitous [6]. Machine Learning (ML) is a key component of modern drug discovery and presents a promising approach to speed up the traditional drug development process and lower costs [7]. There has been a significant adoption of ML across all drug discovery stages and beyond, ranging from target identification to clinical studies [8–10]. The application of machine learning to molecular data, known as Molecular Machine Learning (MolML), has seen extensive use in recent years to accelerate the hit and lead development processes [11]. This includes, but is not limited to, the accurate prediction of binding affinity to (anti-)target proteins, various toxicity-related targets and pathways and physicochemical properties such as solubility [12].

MolML has its beginnings in Quantitiative Structure-Activity Relationship (QSAR) modeling, a methodology that has been applied for over 60 years [13]. These methods are based upon the premise that structurally similar compounds exhibit similar activity, implying that gradual modifications in the structure are accompanied by gradual changes in potency. In molecular datasets, these relationships between the chemical structures of molecules and their target variable are frequently represented as landscapes [14]. Considerable attention has been devoted to investigating the characteristics of these landscapes across different datasets. Discontinuities on the landscape, commonly referred to as Activity Cliffs (ACs) [15], are pairs or groups of structurally similar compounds with large difference in their properties. While they provide a rich source of pharmacological information, they break the “similar molecules have similar properties” principle and hence increase roughness of the landscape. The limitations of ML in presence of these discontinuities have been exposed in several studies [16–20], which conclude that smooth QSAR landscapes are modeled more feasibly by machine learning algorithms.

Over the last decades, domain experts developed various molecular representations for chemical in silico modeling [21, 22]. The most elementary molecular representations are numerical values that quantify molecular properties, ranging from count-based statistics such as the number of atoms to more complex measures including quantum mechanical properties. Initially motivated by the need for efficient database searches, binary fingerprints have become a crucial component in MolML [23]. They indicate the presence or absence of specific substructures within a molecule as a binary vector. Prominent examples include Extended-Connectivity Fingerprints (ECFP) [24], which capture molecular features based on atom connectivity, Molecular Access System (MACCS) keys [25], which encode specific chemical substructures or various pharmacophore descriptors [26], which contain information about the spatial orientation and interactions of a molecule.

In contrast to traditional representation types that depend on predefined rules and manually crafted features, learned representations, also known as embeddings, automatically extract molecular characteristics in a data-driven fashion [27]. These representations are typically produced using the neuron activations of artificial neural networks. Geometric deep learning [28] encompasses Graph Neural Networks (GNNs) [29], which have been adopted in computational chemistry for representation learning on chemical graph structures composed of atoms and bonds [30]. Similarly, deep learning architectures based on the Transformer model [31] have been applied to textual representations of molecules, giving rise to Chemical Language Models (CLMs) [32]. Notably, the most prominent string encodings of molecules include Simplified Molecular Input Line Entry System (SMILES) [33], introduced in 1988, and more recently Self-Referencing Embedded Strings (SELFIES) [34].

Over the past few years, a considerable body of literature has conducted systematic benchmark studies to determine the most promising representation type across datasets [16–18, 20, 23, 35–42]. While these studies sometimes report contradictory results, a general consensus is that no single representation has proven superior, indicating that the effectiveness of representations is highly task-dependent. In addition, representation learning models often show limited performance, specifically in the presence of small dataset sizes, which are common in chemical sciences. Furthermore, pre-training of deep learning representations towards chemical foundation models has not been very effective [43, 44]. In many cases, deep learning representations still have not become the method of choice, and traditional feature vectors are generally favored for their computational efficiency, interpretability and conceptual relevance to the domain.

In this work, we apply Topological Data Analysis (TDA) methods to guide the selection of molecular representations by correlating the topology of the feature space with the generalization capabilities of machine learning models. To better study this connection, we develop a predictive model that learns to link topological descriptors with the generalization error, allowing us to shed light on characteristics of effective representations. In summary, we make the following contributions:

- We find correlations between geometric properties of the feature spaces and machine learning generalizability, thereby elucidating the topological characteristics that contribute to effective representations.
- We develop a predictive model that learns the relationships between topological properties and the generalization error, facilitating the selection of optimal representations and potentially serving as a regularizer for learned representations.
- We perform a comprehensive analysis across 12 datasets, 25 representations, various geometric measures, distance metrics, and models, incorporating hyperparameter tuning and different dataset splitting techniques, resulting in over 100,000 model evaluations.

## Background

### Measuring quantitative structure-activity relationships

QSAR landscapes reveal geometric features of molecular datasets. To capture the magnitude of property change across datasets, several quantitative indices have been developed, which will be introduced in the following. The Structure-Activity Landscape Index (SALI) [45], computes the pairwise similarity between molecules in a dataset and links it to their activity labels. Equation 1 illustrates the calculation of SALI, where $A_i$ and $A_j$ denote the activities of the *i*-th and *j*-th molecule, and *sim*(*i*, *j*) represents the similarity between the two molecules, typically expressed by the Tanimoto coefficient [46].

1 $$\begin{aligned} SALI_{ij} = \frac{|A_i - A_j|}{1 - sim(i,j)} \end{aligned}$$

High SALI values arise from structurally similar molecules that exhibit significant differences in activity, indicating the presence of ACs. These pairwise scores can be visualized in a heatmap, summarizing the local surface and enabling the identification of discontinuities in the data landscape. Structure-Activity Relationship Index (SARI) [47] summarizes the entire QSAR landscape in a single value, which is defined between 0 and 1, with lower values indicating more discontinuous landscapes. Equation 2 details the computation, which incorporates a continuity score ($\text {score}_{\text {cont}}$) and a discontinuity score ($\text {score}_{\text {disc}}$), following a similar rationale as SALI.

2 $$\begin{aligned} SARI = \frac{1}{2}*\text {score}_{\text {cont}} + (1 - \text {score}_{\text {disc}}) \end{aligned}$$

A measure for assessing the modelability of QSAR landscapes is the Modelability Index (MODI). While MODI is only defined for classification problems, the Regression Modelability Index (RMODI) [48] has been developed for regression datasets. As outlined by Eq. 3, the computation is based on the rivality index $RI_j$, a normalized index that measures the label smoothness in the local neighborhood of every data point. Values less than zero imply that the first nearest neighbor of molecule *i* has a similar activity. By summing over all $RI_i < 0$, only non-ACs are considered.

3 $$\begin{aligned} RMODI = \frac{1}{M} \sum _{i=1}^{M} 1, \forall RI_i < 0 \end{aligned}$$

Recently, the Roughness Index (ROGI) [38] and the Roughness Index Extended (ROGI-XD) [36] have been introduced as new measures for capturing the roughness of molecular property landscapes. These indices are inspired by the concept of fractal dimension and show strong correlations with the test error achieved by machine learning models. ROGI captures global surface roughness by measuring the loss in the dispersion of molecular properties as a dataset is progressively coarse-grained.

4 $$\begin{aligned} ROGI = \int _{0}^{1} 2(\sigma _0 - \sigma _t) dt \end{aligned}$$

The method involves complete linkage clustering, substituting property labels with cluster centroids, and computing the standard deviation $\sigma _t$ of the centroids. This sequence is repeated for various distance thresholds *t*. The area under the curve, presented in Eq. 4, yields the ROGI score, with datasets having larger ROGI values resulting in larger model errors. ROGI-XD employs a minor modification in the clustering procedure mitigating the influence of representation dimensionality.

### The shape of data

As demonstrated by QSAR landscape studies, the analysis of the shape of chemical datasets has a long-standing history in computational chemistry. While QSAR analyses are mostly concerned with the shape of property landscapes, little attention has been paid to how molecules are arranged within the feature space. The assumption that high-dimensional data resides on a low-dimensional manifold is the foundation for many machine learning techniques used to analyze such data [49]. Algebraic topology, a subset of mathematics, facilitates the quantitative examination of point clouds and the manifolds they represent. This has given rise to TDA [50], a popular methodology to infer, analyze and exploit geometric shapes. We will provide a condensed introduction in the following section. For a detailed overview, we suggest [51], while more in-depth background can be found in the works of Hatcher [52] and Carlsson [53].

Suppose we have a set of data points $X = \{x_1,...,x_N\} \subset {\mathbb {R}}^d$, each represented as a vector in a *d*-dimensional space. One way to approximate the underlying geometric shape of this point cloud is by connecting nearby points to form simple geometric building blocks, so called simplices, such as points, lines, and triangles. When these simplices are combined, they form a larger structure called a simplicial complex *K*, which can be thought of as a generalized graph that approximates the shape of the data. For computational efficiency, a widely used method is the Vietoris-Rips complex [54], which includes all groups of points that are pairwise within a specified distance $\epsilon$:

5 $$\begin{aligned} VR_{\epsilon }(X) = \{\sigma \subseteq X: \rho (x, y) \le \epsilon \quad \forall x \ne y \in \sigma \} \end{aligned}$$

**Fig. 1:**
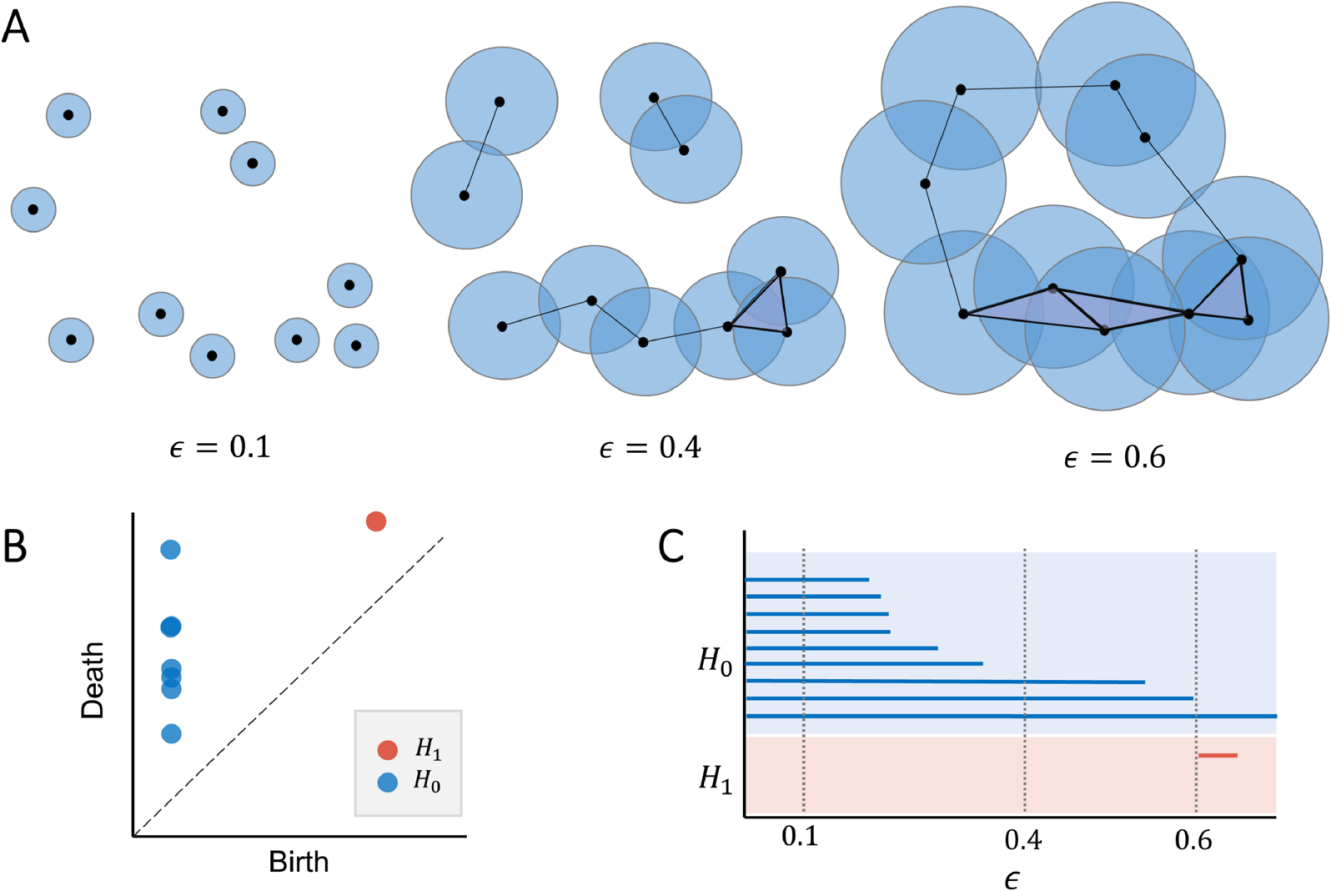
**A** Vietoris-Rips filtration at different scales $\epsilon$. **B** The corresponding persistence diagram for homological dimensions 0 and 1. **C** The corresponding barcode diagram visualizing the lifetimes of persistent features. The cycle in the point cloud is highlighted with red color in the diagrams.

To determine whether two data points are connected in a simplex $\sigma$, we examine if small spheres (called $\epsilon$-balls) centered at each point overlap, using a distance metric $\rho$. We can study the shape of this complex using a tool from algebraic topology called *homology*, which detects structural features at scale $\epsilon$. The $i^{th}$ homology group $H_i$ identifies topological features of dimension *i*, for example connected components and clusters (*i* = 0), cycles (*i* = 1) and voids (*i* = 2). The number of these features is given by the $i^{th}$ Betti numbers of $VR_{\epsilon }$ denoted $\beta _i = dim(H_i)$. As the resolution of the captured topology depends on the scale parameter $\epsilon$, Persistent Homology (PH) tracks the topological features across different scales. At scale $\epsilon = 0$, $VR_0(X)$ is a collection of 0-dimensional simplices ($\beta _0 = |X|$). As the scale increases, the $\epsilon$-balls of the points overlap, forming higher-dimensional simplices, which results in a richer topology. This is known as *Vietoris-Rips filtration* (see Fig. 1A) and is mathematically defined as a nested sequence of simplicial complexes $K_0 \subseteq K_1 \subseteq ... \subseteq K_m = K$ representing the growth of *K* as the scale $\epsilon _0 \le \epsilon _1 \le ... \le \epsilon _{m}$ is being changed.

During filtration, these topological features can be created or destroyed (by merging simplices), which is tracked by PH. The evolution of features can be visualized with different diagram types. In the persistence diagram in Fig. 1B, each point $(b_i^j, d_i^j) \in {\mathbb {R}}$ corresponds to the creation (birth) and destruction (death) of a *i*-dimensional feature. Points close to the diagonal are considered noise and points further away are significant features, which persist over a wide range of scales. The difference $d_i^j - b_i^j$ corresponds to the topological invariant *persistence*, which expresses the duration of a feature, as illustrated in the barcode diagram in Fig. 1C. Based on the lifetimes of the topological features, more descriptive properties can be derived, which will be addressed in the Methods section.

### Generalization bounds and the role of topology

The concept of *modelability*, more commonly referred to as *generalizability* in machine learning, quantifies a model’s performance in terms of empirical risk minimization, i.e., how effectively a model can generalize from training data to unseen data. A number of studies have attempted to understand generalization in ML, proposing various upper bounds on the test error, generally known as *generalization bounds* [55]. To link the intrinsic structure of machine learning models to their generalization capabilities, researchers have developed several complexity measures. These include both theoretically motivated measures like the VC-dimension [56] and empirically motivated measures such as the sharpness of minima in deep learning optimization trajectories [57]. Additionally, measures based on PH have been used to predict the generalization of neural networks [58], and recent studies have correlated the intrinsic dimension with test accuracy [59].

In this study, we incorporate multiple data-dependent generalization measures, which are summarized in the following. In the realm of PH a novel geometry-based generalization bound known as the Persistent Homology Dimension $dim_{PH}^i$ [60] has recently found several applications [61]. It is motivated by fractal dimensions, which were introduced by Hausdorff and others to measure the complexity of spaces [62]. Intuitively, $dim_i^{PH}$ is determined by how the lengths of the PH intervals for increasing sample sizes vary for a specific homological dimension *i*. Higher values suggest a more complex topology across scales, as the total persistence (sum of lifetimes) grows rapidly with sample size. Let *X* be a bounded subset of a metric space and define the $\alpha$-weighted sum of the length of persistence intervals, given by their birth *b* and death *d* times as:

6 $$\begin{aligned} E_i^\alpha =\sum _{(b,d) \in PH_i(x)} (d-b)^\alpha \end{aligned}$$

Then $dim_i^{PH}$ for the Vietoris-Rips complex of *X* corresponds to the smallest $\alpha$ for which $E_i^\alpha$ is uniformly bounded for all subsets of *X*:

7 $$\begin{aligned} dim_i^{PH}(X) = \inf \{\alpha : \exists C \quad s.t. \quad E^i_\alpha (x) < C \quad \forall x \subset X\} \end{aligned}$$

In recent years, Intrinsic Dimension (ID) has found broad usage as an essential geometric property of data representations [63], but has not yet been studied in the context of molecular representations. ID reflects the minimum number of variables needed to capture the essential structure of the data. We employ two techniques for estimating ID that have emerged over the years. TwoNN estimates the global intrinsic dimension based on the distances to the two nearest neighbors of every point [64].

8 $$\begin{aligned} dim_{\text {TwoNN}} = \frac{\log {(1-F(\mu ))}}{\log {(\mu )}} \end{aligned}$$

In Eq. 8, $\mu$ represents the ratio of the two shortest distances and $F(\mu )$ the cumulative distribution of these ratios sorted in an ascending order. $dim_{\text {TwoNN}}$ corresponds to the slope of a line fitted through the origin and $(\log {(\mu )}, -\log {(1-F(\mu )) | i=1,...,N})$. Another widely utilized estimate for ID is derived from eigenvalue analysis, commonly referred to as $dim_{PCA}$ due to its foundation in Principal Component Analysis (PCA). In this study, we follow the computation of Fukunaga et al. [65], which involves computing eigenvalues of the covariance matrix for each local region.

## Methods

**Fig. 2:**
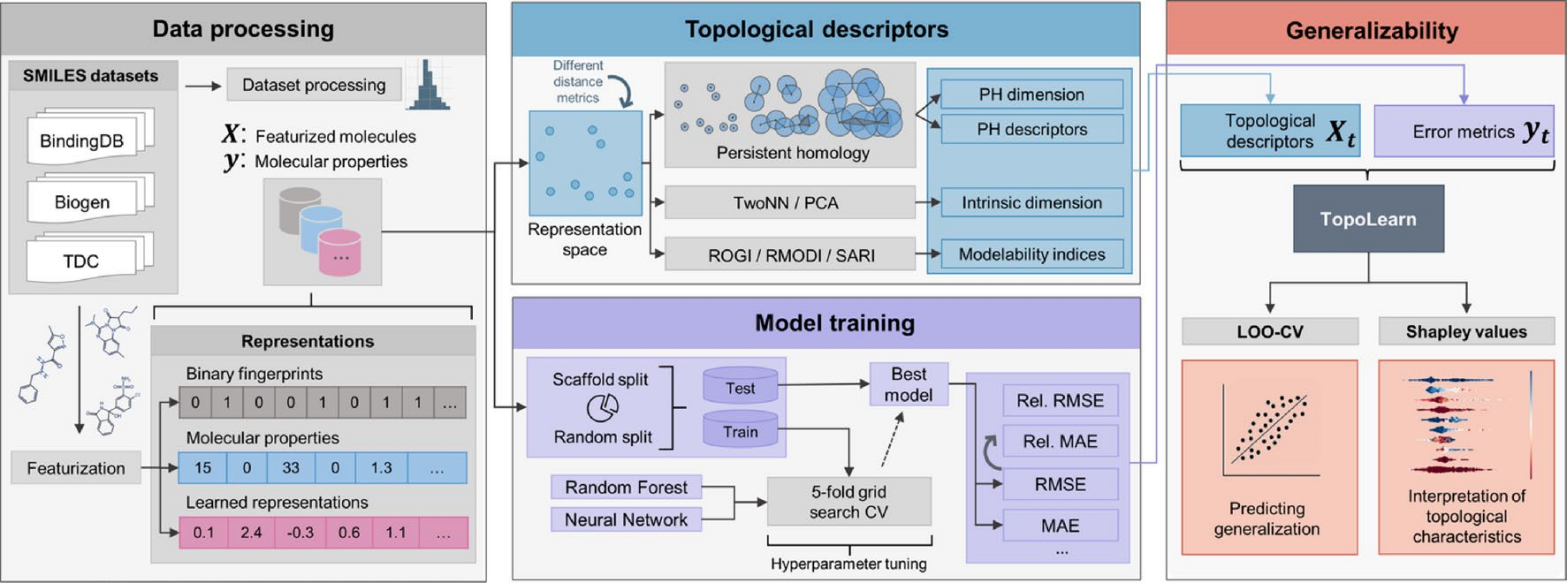
Overview of the TopoLearn framework. **Left:** Molecular data is featurized and preprocessed, resulting in a collection of machine learning datasets. **Top:** Topological descriptors and metrics are computed from the arrangement of the data in the feature space. **Bottom:** Machine learning models are optimized through hyperparameter tuning for each dataset and the best model’s error metrics are calculated. **Right:** TopoLearn is trained to predict the error metrics based on the topological descriptors. The model’s performance is evaluated and interpreted using Shapley values

We carry out an extensive study to explore the influence of representation space topology on the effectiveness of molecular machine learning, as illustrated in Fig. 2. We preprocess and featurize 12 molecular datasets and use 25 different molecular representations for model training. Additionally, we compute the topological characteristics of the feature spaces in terms of PH descriptors, ID estimates and modelability indices. We combine the test errors with these topological descriptors to create a new dataset, which we use to fit TopoLearn, a model capable of predicting machine learning performance.

### Datasets

**Fig. 3:**
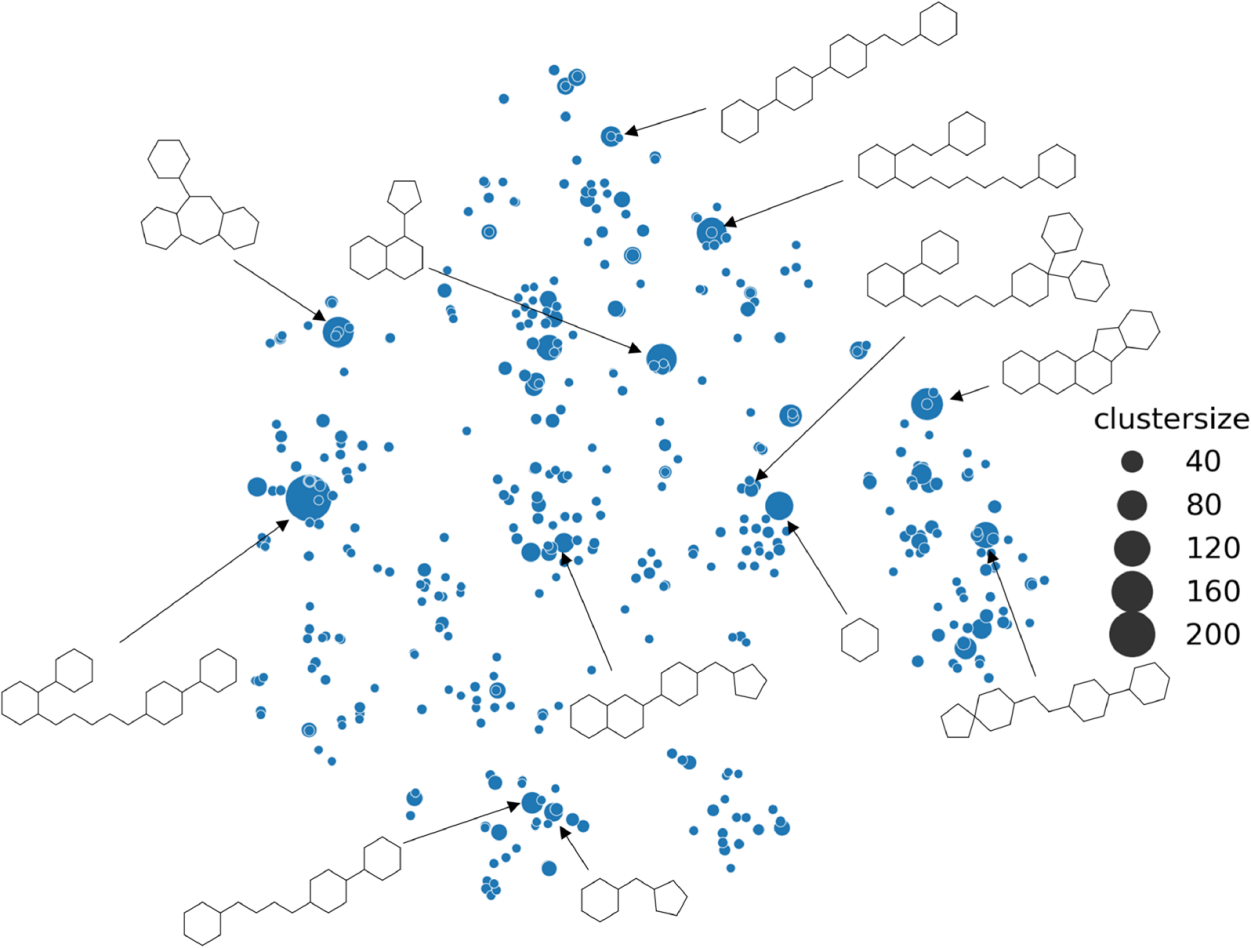
T-SNE projection of reduced graph Bemis-Murcko scaffolds for ADRA1A dataset

MolML benchmark datasets have faced criticism for their quality issues and lack of pharmaceutical relevance [16, 66]. Therefore, we concentrate on relevant real-world regression datasets from different biochemical domains as shown in Table 1. We use protein-ligand binding affinity datasets, which measure the inhibition constant $K_i$), sourced from BindingDB [67]. Specifically, we include the following datasets: ADRA1A, measuring the binding interactions of molecules with the alpha-1A adrenergic receptor; ALOX5AP, providing binding information on the arachidonate 5-lipoxygenase-activating protein; ATR, containing binding data for the ataxia telangiectasia and Rad3-related protein; DPP4, comprising biochemical data of human dipeptidyl peptidase-4 inhibition; JAK1 and JAK2, covering interactions between ligands and the Janus Kinase proteins; MUSC1 and MUSC2, including binding data for subtypes of muscarinic receptors; and KOR, a dataset for kappa-type opioid receptor binding. We also include a physicochemical dataset from Therapeutics Data Commons (TDC) [68] which contains the lipophilic properties of various molecules. Additionally, we use well-curated datasets from Biogen [66]; a physicochemical dataset measuring aqeuous solubility at pH 6.8 and an Absorption, Distribution, Metabolism, and Excretion (ADME) dataset reporting the intrinsic clearance in human liver microsomes. In supplementary file 1, we present a range of dataset statistics and details on molecular diversity, as shown by Fig. 3.

In the preprocessing step we converted all SMILES strings to the SDF format and excluded compounds with missing stereochemical information using RDKit [69]. The remaining compounds were neutralized, dearomatized, and had salts removed using JChem standardizer (version 24.3.2). Afterwards, all compounds were ionized to pH 7 with MoKa (version 3.2.3). Canonical SMILES were then calculated with RDKit from the resulting structures, serving as the basis for subsequent featurization. We also explored label standardization for the ML models, which had no significant influence on the results. For our analysis, we evaluated model performance across various dataset sizes by randomly sampling without replacement, ranging from 500 to 2500 molecules in increments of 500.

**Table 1:** Molecular datasets, their size after preprocessing, the origin of the data and their minimum and maximum label values

Dataset name	# Molecules	# Removed	Data type	Source	Min	Max
ADRA1A	2847	127	Inhibition	BindingDB	− 4.61	12.74
ALOX5AP	3067	127	Inhibition	BindingDB	− 2.30	9.21
ATR	4249	617	Inhibition	BindingDB	− 1.97	10.87
DPP4	3345	1	Inhibition	BindingDB	3.0	10.92
JAK1	4504	1316	Inhibition	BindingDB	− 3.44	12.84
JAK2	1449	2	Inhibition	BindingDB	− 3.51	15.76
KOR	1685	91	Inhibition	BindingDB	− 5.47	16.31
MUSC1	2614	746	Inhibition	BindingDB	− 4.84	16.12
MUSC2	1110	0	Inhibition	BindingDB	− 3.51	12.90
LIPO	3495	1	Physicochemical	TDC	− 1.5	4.5
SOL	2173	91	Physicochemical	Biogen	− 1.0	2.18
HLMC	3087	110	ADME	Biogen	0.67	3.37

### Representations

We concentrate on unimodal representations derived from the 2D chemical structure to ensure that all representations are based on the same information. We categorize the molecular representations into binary fingerprints, molecular properties, and learned representations to take the diverse underlying feature spaces into account.

**Table 2:** Learned molecular representations

Representation name	Architecture	# pre-training samples	Dimensionality
ChemBERTa [70]	Transformer	77M	384
ChemGPT [71]	Transformer	19M	256
SafeGPT [72]	Transformer	1.1B	768
MoLFormer-XL [73]	Transformer	110M	768
Graphormer [74]	GNN + Transformer	4M	768
GIN [75]	GNN	500K	300
Mol2Vec [76]	RNN	20M	300

The learned representations originate from different datasets that vary in size and molecular distribution, as detailed in Table 2. While the data used to learn these representations is crucial for their quality, it is challenging to account for all influencing factors, such as model architecture, training data size, optimization algorithm, or embedding dimension. In this study, we focus solely on the resulting topological configuration, without considering the methods used to create the features. MolFeat [77] and HuggingFace [78] were used to generate embedding vectors for the SMILES data. We include the pre-trained language models ChemBERTa [70], ChemGPT [71], SafeGPT [72] and MolFormer-XL [73], which are trained on different textual descriptions of molecules. We also include Mol2Vec [76], which is trained using an unsupervised machine learning approach inspired by natural language processing. In addition, we use the pre-trained Graph Isomorphism Network (GIN) [75] and Graphormer [74], which are architectures based on GNNs. Furthermore, we experimented with a random feature vector as a ’worst case’ baseline, as done for ROGI-XD [36]. However, we observed that it significantly influenced the correlation analysis, leading us to decide to discard it.

**Table 3:** Binary fingerprints

Representation name	Fingerprint type	Dimensionality
ECFP4 [24]	Circular	1024
MAP4 [79]	Circular	1024
RDKit [69]	Path-based	1024
AVALON [80]	Path-based	1024
MACCS [25]	Structural key	166
KR [81]	Structural key	4860
PubChem [82]	Structural key	881
CATS2D [83]	Pharmacophore	189
Pharm2D [84]	Pharmacophore	1024
EState [85]	Other	79

For binary fingerprints (Table 3), we use the circular fingerprints ECFP4 [24] and MAP4 [79], which encode molecular substructures by considering atoms and their neighbors up to a certain radius. Further, we include the RDKit fingerprint [69] and AVALON [80], both of which are path-based fingerprints that encode the presence or absence of specific substructures defined by paths of atoms and bonds within the molecule. We investigate structural keys including MACCS [25], Klekota-Roth (KR) [81] and PubChem [82], which utilize predefined sets of substructure patterns to represent molecules. Additionally, we use the pharmacophore fingerprints CATS2D [83] and Pharm2D [84], which capture the spatial arrangement of pharmacophoric features in two dimensions. Besides these types, we incorporate EState [85] fingerprints, which encode information about the electronic and topological state of atoms within a molecule. We used MolFeat [77], RDKit [69] and the MAP4 Python package to generate the bit-vectors.

**Table 4:** Molecular properties computed with AlvaDesc [86]

Representation name	Description	Dimensionality
TOPO	Connectivity and arrangement of atoms	74
RingDesc	Number and type of ring structures	35
FGCount	Presence of specific functional groups	153
2DAP	Spatial relationships between pairs of atoms	1596
ConstIdx	Basic structural properties	50
WalkPath	Connectivity and paths within a molecule	46
MolProp	Descriptor used in [36]	14
Combined	Concatenation of all molecular descriptors	1992

In addition, we used AlvaDesc [86] and PaDEL [85] to compute several molecular properties, including descriptors of ring structures, functional groups, walk counts and path lengths, atom pairs and more as detailed in Table 4. We also concatenate all computed properties into a joint representation called ’Combined’.

### Model training

Experiments have demonstrated that neural networks tend to favor smooth solutions [87]. This can limit their performance when dealing with irregularities, such as ACs in chemical datasets. Consequently, tree-based ensemble models are often preferred over neural networks, particularly in the domain of chemical sciences. Given the critical role of model selection, we train both a random forest and a fully-connected neural network model on all fixed molecular representations across all datasets using scikit-learn [88]. We perform a hyperparameter optimization using 5-fold grid search cross-validation for both scaffold and random dataset splitting, resulting in a total of 104,028 training runs. For scaffold splitting, we compute Bemis-Murcko scaffolds [89] for each molecule and perform a group split on the scaffolds.

Variations in dataset difficulty can lead to different ranges of error values. To make representation performance comparable across datasets, we normalize each error metric $E_i$ within each dataset $d \in D$ to the range [0, 1], as detailed by Eq. 9. Further details on the cross-validation procedure and the chosen error metrics are provided in Appendix A.

9 $$\begin{aligned} E_{i, \text {rel}}^d = \frac{E_{i}^d - E_{\text {min}}^d}{E_{\text {max}}^d - E_{\text {min}}^d} \quad \forall d \in D \end{aligned}$$

### Topological features

We use the Python package ripser [90] to compute the Vietoris-Rips filtration for each dataset and representation for homological dimensions $i \in \{0, 1\}$. We remove the special persistence interval [0, $\infty$] and use the remaining features to compute various one-dimensional summary statistics, as shown in Table 5. We compute the *i*-th Betti number $\beta _{i}$, which quantifies the number of connected components ($i=0$) and the number of loops in the space ($i=1$). Additionally, we normalize these values by the total number of data points in the dataset. For *m* computed persistent features $\text {PH}_i$, we mathematically define the lifetime of the *j*-th feature in the persistence diagram as $\Delta _i^j = d_i^j - b_i^j$ and the midlife of the *j*-th feature as $\mu _i^j = \frac{b_i^j+d_i^j}{2}$, where *b* and *d* represent the birth and death times of the feature. Further, we define the normalized lifetime as $p_i^j = \frac{\Delta _i^j}{\sum _{k=1}^{m}\Delta _i^k}$ and the normalized midlife as $\nu _i^j = \frac{\mu _i^j}{\sum _{k=1}^{m}\mu _i^k}$. By applying the aggregators $agg \in {\{min, max, avg, std, sum\}}$ we compute topological descriptors of the lifetimes $L_i$ and the midlifes $M_i$ along with their normalized variants. Furthermore, we use the Python package giotto-tda [91] to compute the persistence entropy $E_i$ [92], a measure for persistence variability, for $i \in \{0, 1\}$.

**Table 5:** Persistence diagram descriptors for homological dimension $i \in \{0, 1\}$

Symbol	Topological descriptor	Mathematical description
$\beta _{i}$	Betti number	$\text {dim}(H_i)$
$\beta _{i}^{norm}$	Normalized Betti number	$\frac{\beta _{i}}{n}$
$L_i^{agg}$	Lifetime statistics	$agg(\{\Delta _i^j\}) \quad \forall \Delta _i^j \in \text {PH}_i$
$L_i^{agg}$	Normalized lifetime statistics	$agg(\{p_i^j\}) \quad \forall p_i^j \in \text {PH}_i$
$M_i^{agg}$	Midlife statistics	$agg(\{\mu _i^j\}) \quad \forall \mu _i^j \in \text {PH}_i$
$M_i^{agg}$	Normalized midlife statistics	$agg(\{\nu _i^j\}) \quad \forall \nu _i^j \in \text {PH}_i$
$E_i$	Persistence entropy	$-\sum _{p_i^j \in \text {PH}_i} p_i^j log(p_i^j)$

The computed topological features are sensitive to the underlying distance metric *d*, as they emerge entirely from pairwise distances. We investigate the influence of *d* and conduct all analyses using various distance metrics, as elaborated in Appendix B.1. We also analyze the effect of computing the topological features on all data versus only the train data used in cross-validation, as described in B.6.

### Dimension estimates

We compute the *i*-th Persistent Homology Dimension $dim_i^{PH}$ for every combination of representation, dataset, distance metric, sample size and $i \in \{0, 1\}$. Following the approach of [61], we sample *n* points $X_n$ from *X*, where $n \in \{100, 150, 200, \ldots , 2000\}$, in steps of 50, and compute the sum of persistence lifetimes $E^i_\alpha (X_n)$. The empirical slope *m* between the logarithmic values of $E^i_\alpha$ and *n* provides an upper bound estimate through $dim_i^{PH} + \epsilon \le \frac{m}{1-\alpha }$. We fit a linear regression to estimate the slope *m* and use $\frac{\alpha }{1-m}$ as estimate of $dim_i^{PH}$, where $\alpha =1.0$ in our experiments. Further, we estimate the ID using scikit-dimension [93] and compute $dim_{\textit{TwoNN}}$ and $dim_{\textit{PCA}}$ for all training configurations.

### TopoLearn

As illustrated in Fig. 2, we use the previously computed topological descriptors along with the results of the model training to construct a new dataset $(X_t, y_t)$, which links topology with machine learning performance. Initially, we examine simple correlations between model errors and topological features. However, we anticipate that employing a non-linear model will yield a stronger relationship compared to individual scores. We conduct hyperparameter tuning and train a random forest regressor to predict the normalized root mean squared error (RMSE). This model is chosen for its robustness and its capacity to handle missing values effectively. Since the normalized RMSE is constrained to the interval [0, 1], we clip any model predictions that fall outside this range. The TopoLearn dataset contains 2550 samples for both scaffold and random splitting along with 66 features. We incorporate all hyperparameters, the sample size, feature dimensionality and distance metric as additional (potentially one-hot encoded) features in the model to account for their influence and prevent spurious correlations.

**Fig. 4:**
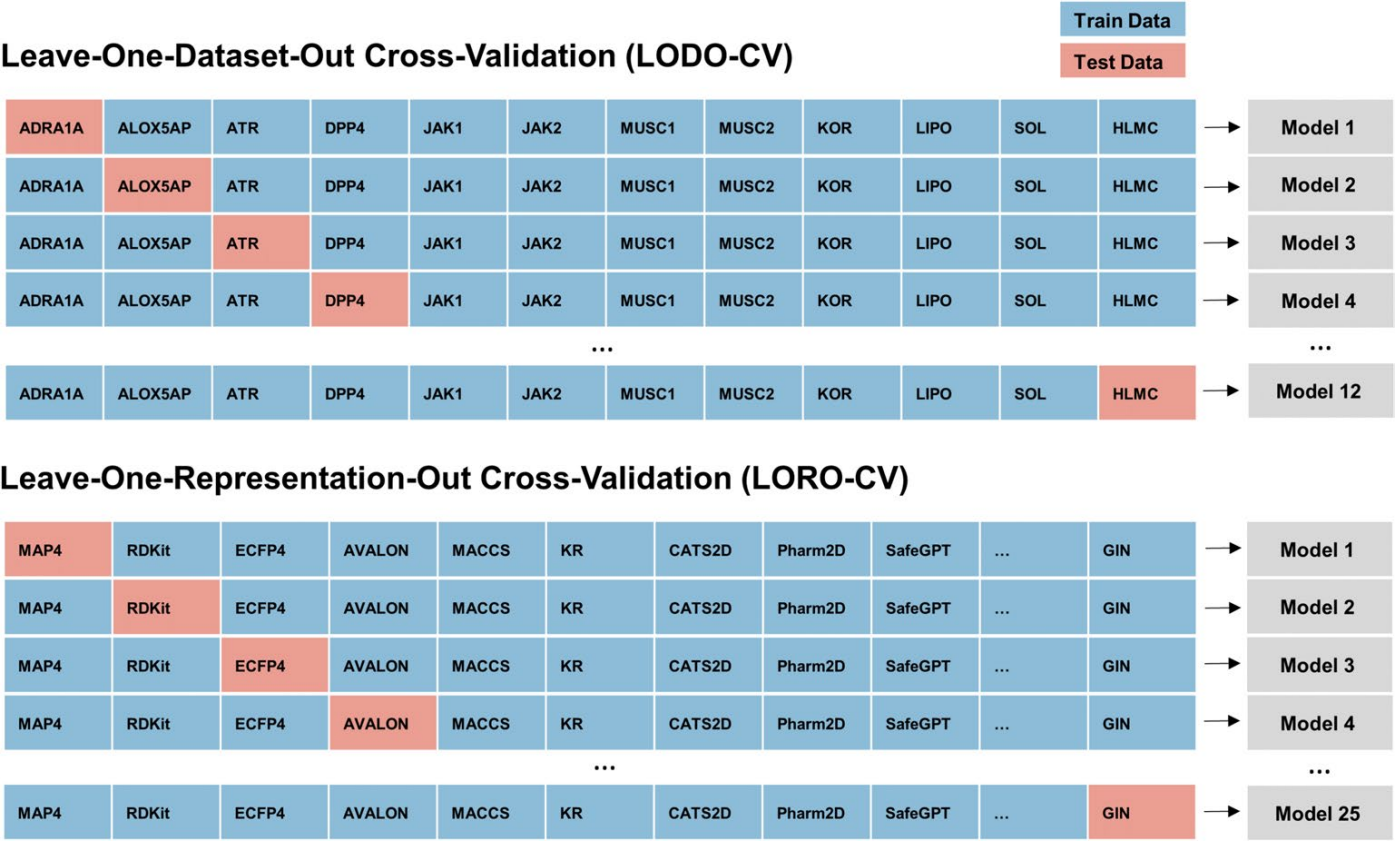
LODO-CV and LORO-CV cross-validation schemes

To evaluate the performance of TopoLearn on unseen data, we employ two leave-one-out validation strategies, as depicted in Fig. 4. First, we perform a leave-one-dataset-out cross-validation (LODO-CV), which aims at evaluating the model’s capability to predict representation errors on entirely new datasets. Secondly, we perform a leave-one-representation-rut cross-validation (LORO-CV) to assess how TopoLearn predicts the error of novel representations. We perform these experiments using various dataset splitting strategies, feature sets, and distance metrics to ensure validity across all configurations. We aggregate the predictions of each holdout fold to obtain predictions for the entire dataset and compute the correlation with the actual model errors. Additionally, we construct feature summaries using the Python library SHAP [94], which enables us to derive feature importances and their effects on predictions, while accounting for confounding factors. For the random forest models we make use of the TreeExplainer implementation. Furthermore, we use the implementation of [38] and [36] to compute ROGI-XD, RMODI and SARI and use these metrics as baselines for comparison with TopoLearn.

## Results

### Analysis of representation performance across datasets

**Fig. 5:**
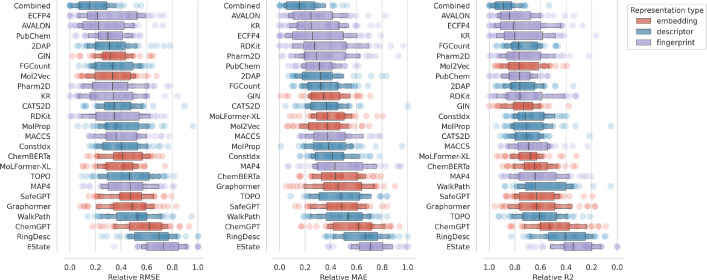
Normalized test error distribution per representation across all datasets for sample sizes $\ge$ 1000 on randomly split data

We are interested in the most effective representations across all experiments and therefore aggregate all model errors for different error metrics. Figure 5 ranks the representations by their median normalized prediction error on randomly split datasets. We observe comparable outcomes for the scaffold-split data, with the corresponding figures presented in Appendix B.3. The representation combining all molecular property descriptors consistently achieves low errors and shows significantly lower variance compared to other representations. This is followed by the fingerprint representations AVALON and ECFP4, which are widely used molecular fingerprints. Despite their overall performance, the large variance in error distributions indicates that these descriptors are not universally superior. Moreover, we observe that learned representations are not among the top-ranked, which is in line with the results of the studies referenced in the introduction.

### Persistent homology lifetimes

**Fig. 6:**
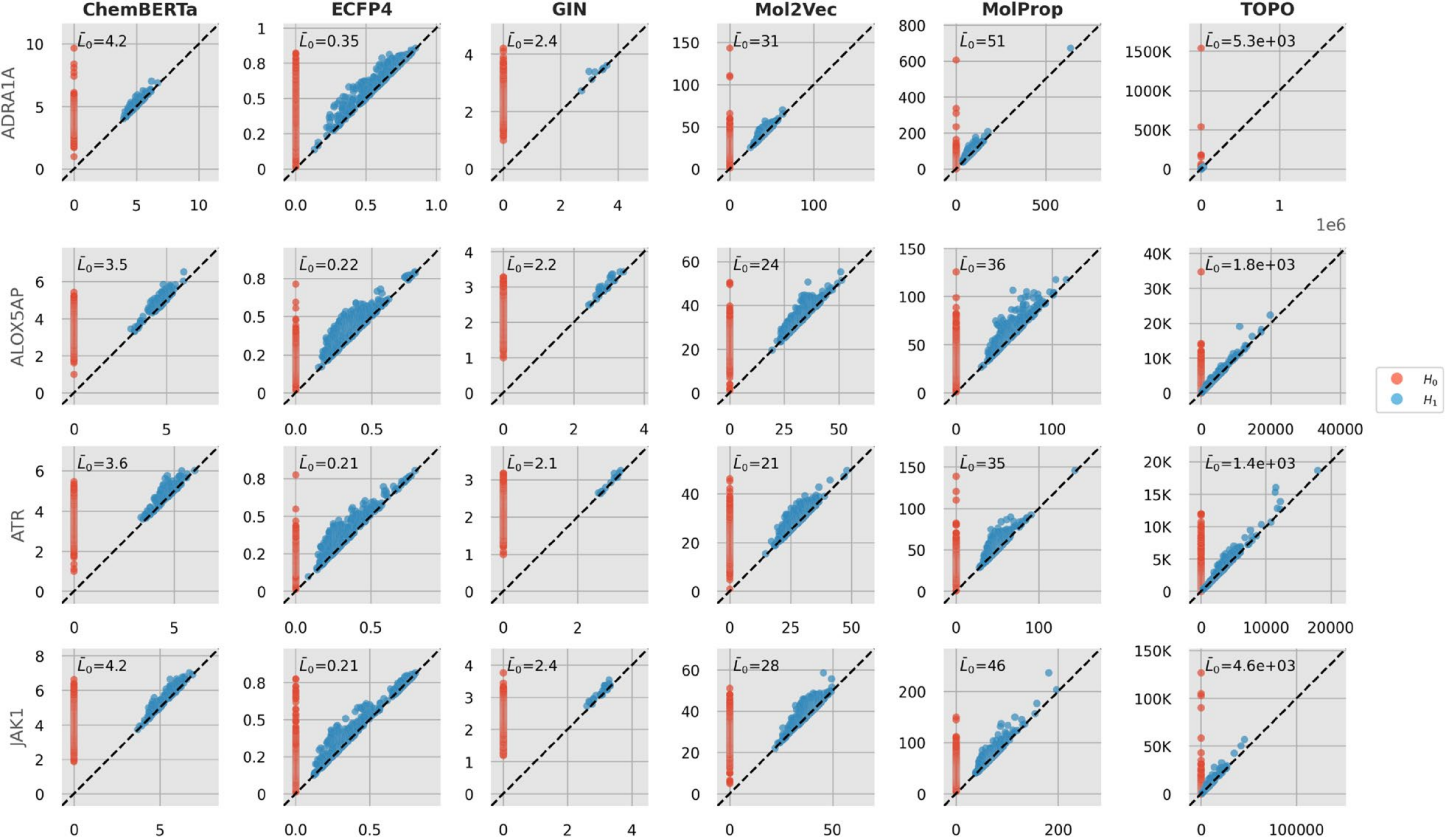
Persistence diagrams for selected datasets and representations. Red points are features for homological dimension 0, blue points are features for homological dimension 1. ${\hat{L}}_0$ denotes the mean lifetime of 0-dimensional features

**Fig. 7:**
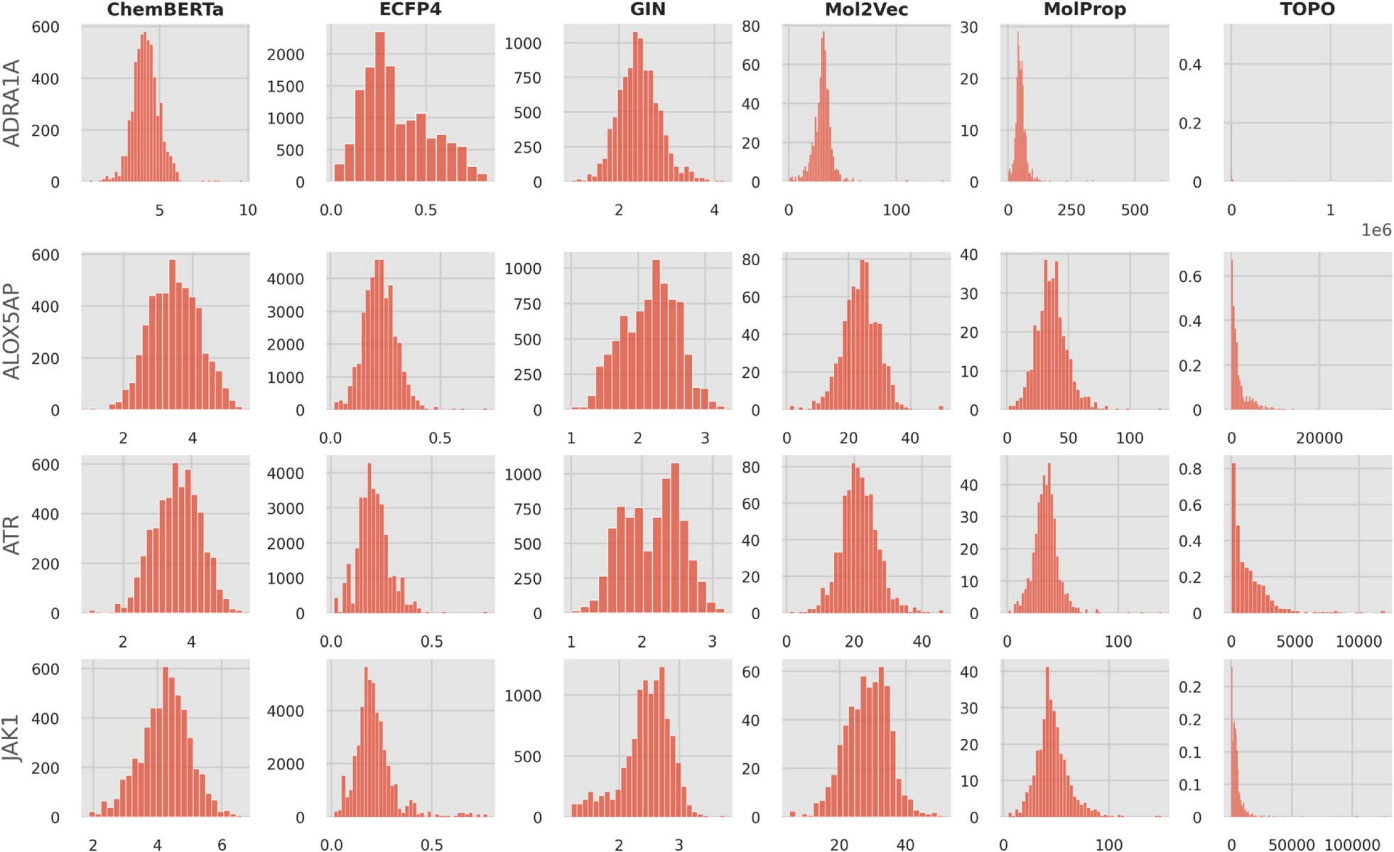
Persistence lifetime histograms for selected datasets and representations. The y-axis corresponds to the absolute number of observed features

We analyze the properties of persistence diagrams computed for the different molecular machine learning representations to gain an understanding for their topological characteristics. An example of persistence diagrams for different representation types is provided in Fig. 6. Additional diagram visualizations can be found in supplementary file 1. The diagrams reveal that binary fingerprint representations produce significantly shorter persistence intervals along with more high-dimensional features in homological dimension 1. This observation can be explained by the underlying Jaccard distance measure, which ranges from 0 to 1. Besides the choice of distance metric, the feature ranges of each representation significantly impact the magnitude of topological features. This effect is particularly evident in molecular descriptors, which often exhibit very lengthy features. Further, we find large variations in higher-dimensional topological features, suggesting that there are differences in topological complexity. We investigate the distribution of persistence lifetimes *L* for homological dimension 0 in more detail as shown by Fig. 7. We find that the lifetime distributions vary across representations and datasets, demonstrating that each combination leads to characteristic features. Notably, despite choosing the same number of samples for the datasets in the figures, the number of features differs for each representation.

### Correlation and feature influence analysis

**Fig. 8:**
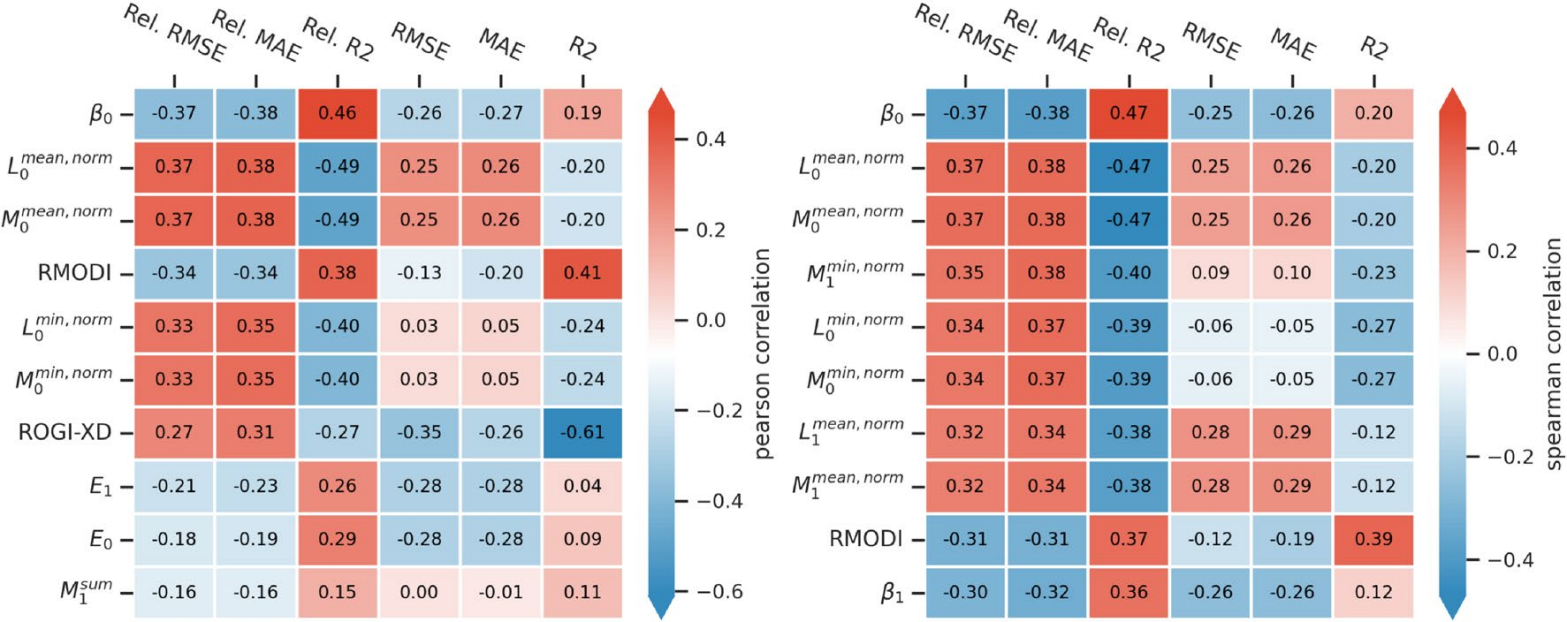
Pearson correlation and Spearman rank correlation coefficient of the most correlated topological features with different error metrics, computed across all randomly-split datasets and representations

We perform a statistical correlation analysis to investigate if there is a direct relationship between topological properties and test error. While such measures are unlikely to fully explain the performance of certain representations, given the importance of strong features, our goal is to shed light on parts of this effect by analyzing the topological configuration. We compute the Pearson correlation and Spearman rank correlation coefficient to identify relationships between topological properties and the relative model error. Figure 8 summarizes the properties with the highest correlations. The significance of all correlations was evaluated using a two-tailed hypothesis test with sample sizes of 714 (embeddings), 918 (fingerprints), and 918 (descriptors). Across all representations, persistent homology descriptors show the highest correlations with the model test errors. Specifically, the Betti numbers ($r=-0.37, p<0.001$), normalized mean lifetimes ($r=0.38, p<0.001$) and normalized mean midlifes ($r=0.38, p<0.001$) of homological dimension 0 show significant correlations with the mean absolute errors of the machine learning models for both correlation measures. Notably, the various descriptors of persistence lifetimes are highly correlated with each other, and other aggregation methods exhibit similar patterns. In addition, we observe strong negative correlations between ROGI-XD and the coefficient of determination $R^2$ ($r=-0.61, p<0.001$) as well as between RMODI and $R^2$ ($r=0.41, p<0.001$). This highlights the importance of the chosen error metric. Moreover, we find that ROGI-XD has a higher correlation with unnormalized error metrics, which suggests that it is a good indicator for the absolute scale of errors but less suitable for comparing representations within a dataset.

As described in Appendix B.1, the underlying distance metric affects the computation of topological properties. Consequently, we perform individual correlation analyses for each representation type, which are presented in Appendix B.4 (See Figures 19, 20, 21). The results of the correlation analysis suggest that there are connections between the topological descriptors and the model errors, which builds a foundation for non-linear modeling with TopoLearn.

### Predicting generalization with TopoLearn

We carry out a leave-one-dataset-out cross-validation evaluation by training TopoLearn on data from $k-1$ datasets and predicting the normalized test error on all training runs of the $k^{th}$ dataset. In addition to analyzing all representations collectively, we also evaluate the results individually for each representation type. We investigate two variants of our model: TopoLearn (C), which is trained using all persistent homology descriptors, dimension estimates, and control variables (such as sample size, dimensionality or hyperparameters) as features, and TopoLearn (PH), which is trained exclusively on persistent homology descriptors. This approach enables us to isolate and analyze the impact of the additional variables within the model. TopoLearn (C) will be used solely for analysis purposes and then discarded, as some inputs will be unavailable during inference. The results are summarized in Table 6 for randomly split data, along with a comparison with the baseline modelability indices. The table for scaffold split data is available in Appendix C(See Table 10, 11). We report the Pearson correlation coefficient of all cross-validation runs $r_{cv}$ and the correlations on the aggregated data $r_{a}$. During the computation of SALI, we encountered NaN-values, likely due to the use of the Euclidean distance metric. Consequently, we provided the SALI results only for binary fingerprints using the Jaccard distance.

**Fig. 9:**
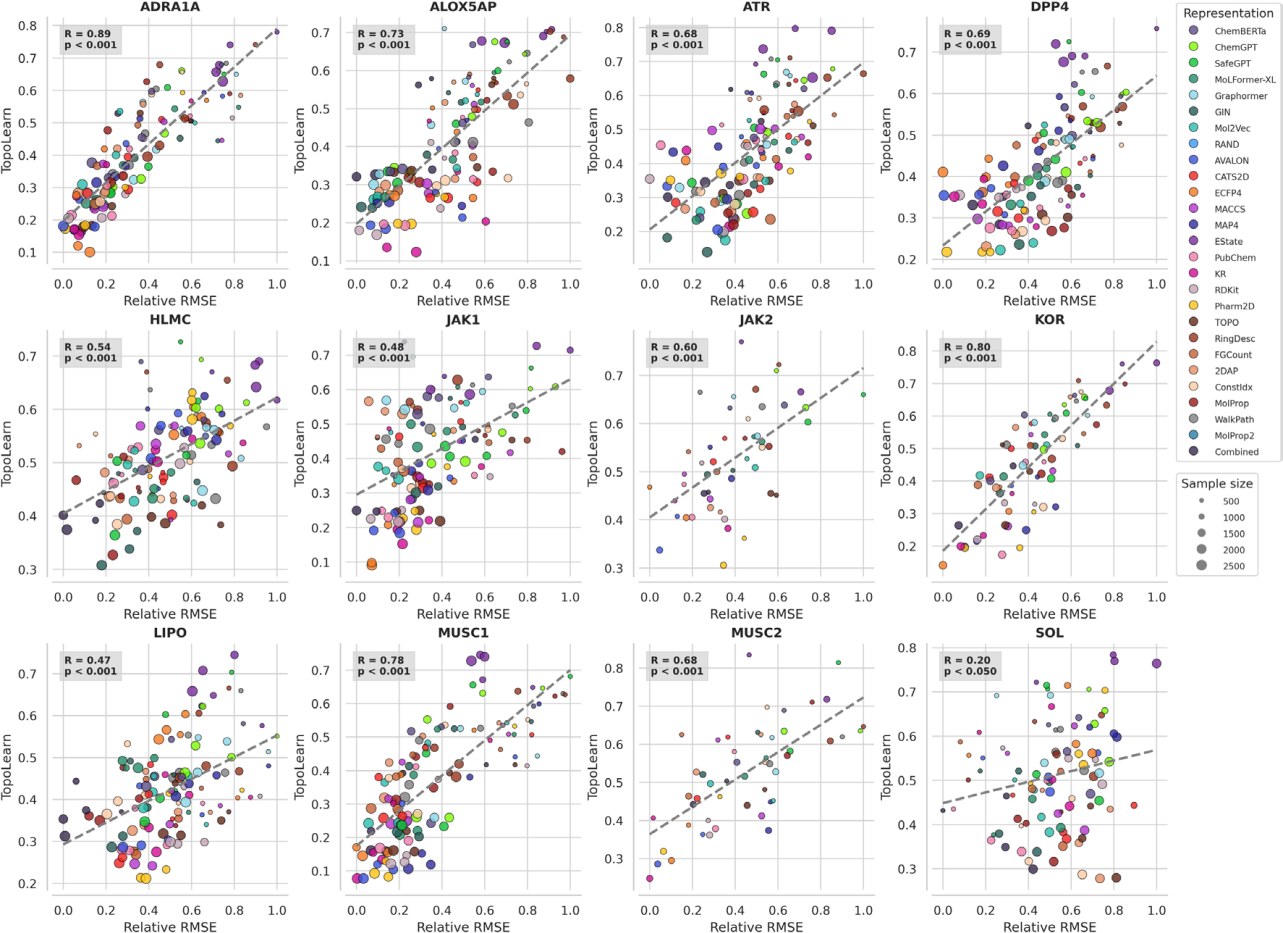
TopoLearn leave-one-dataset-out cross-validation scatterplots on randomly split data

For both splitting techniques, TopoLearn (PH) has significantly higher average correlations ($r=0.62, p<0.001$) compared to all other modelability indices. A paired t-test comparing our method to the second-best baseline revealed a statistically significant improvement (t = 10.35, p $<0.001$), providing strong evidence of the superiority of our approach across the evaluated datasets. As shown in Fig. 9, the correlation strength varies across datasets, which is reflected by the large variance of 0.18. We observe strong correlations for all binding datasets (r $\in$ [0.48; 0.89]) and lower correlations for the solubility ($r=0.2, p<0.05$) and lipophilicity ($r=0.47, p<0.001$) data. Additionally, the scatterplots demonstrate the influence of sample size, where larger datasets tend to have lower errors. We observe the strongest correlations ($r=0.68, p<0.001$) for TopoLearn (C), suggesting that parts of the test error can be explained by other variables, such as sample size. This will be further discussed in the subsequent section. In addition, we find that all evaluated generalization measures perform best on fingerprint representations and worst on molecular descriptors.

**Table 6:** Pearson correlation coefficient between generalization measures and the relative RMSE. $r_{cv}$ denotes the coefficient for leave-one-representation-out cross-validation on random splits and $r_{a}$ the coefficient measured on all aggregated predictions

	All		Embeddings		Fingerprints		Descriptors	
	$r_{cv}$	$r_{a}$	$r_{cv}$	$r_{a}$	$r_{cv}$	$r_{a}$	$r_{cv}$	$r_{a}$
TopoLearn (C)	0.68 ± 0.16	0.68	0.67 ± 0.18	0.68	0.69 ± 0.17	0.74	0.67 ± 0.17	0.62
TopoLearn (PH)	**0.62** ± **0.18**	**0.63**	**0.65** ± **0.18**	**0.66**	**0.67** ± **0.17**	**0.73**	**0.49** ± **0.26**	**0.46**
ROGI-XD	0.28 ± 0.16	0.28	0.32 ± 0.36	0.16	0.46 ± 0.23	0.41	0.06 ± 0.24	0.14
RMODI	-0.36 ± 0.21	− 0.35	− 0.24 ± 0.26	− 0.13	− 0.60 ± 0.20	− 0.55	− 0.17 ± 0.14	− 0.16
SARI	–	–	–	–	-0.23 ± 0.16	0.24	–	–

**Fig. 10:**
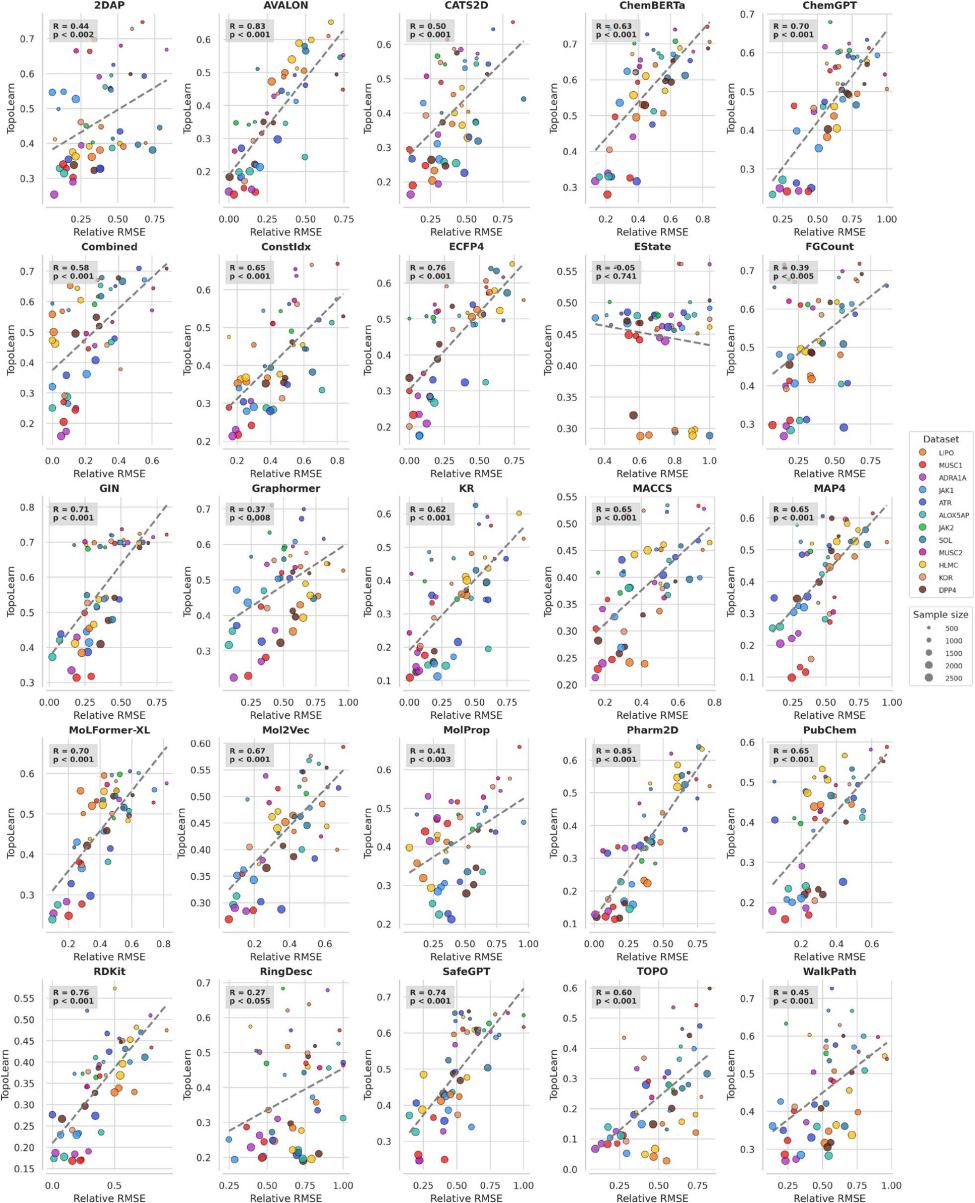
TopoLearn leave-one-representation-out cross-validation scatterplots on randomly split data

Following the same approach as the LODO analysis, we implement LORO-CV to evaluate TopoLearn’s ability to predict the relative test error for unseen representations. The results are summarized in Table 7 using the same notation as before. A paired t-test comparing our method to the second-best baseline revealed a statistically significant improvement (t = 6.91, p $<0.001$), providing strong evidence of the superiority of our approach across the evaluated representations. We observe that the overall correlations are slightly lower compared to the LODO analysis, indicating that predicting the performance of novel representations is more challenging. TopoLearn (PH) consistently outperforms the other measures, achieving an average correlation of $r=0.58$ $(p<0.001)$. The results improve when analyzing embeddings ($r=0.65, p<0.001$) and fingerprints ($r=0.62, p<0.001$) separately, but are less significant for descriptors alone ($r=0.46, p<0.001$). High variances across datasets are also evident for LORO, as depicted in Fig. 10. Detailed analysis reveals that the EState and MolProp representations do not produce significant results, while binary fingerprints achieve the highest correlations. The best results are again observed with additional variables for TopoLearn (C) ($r=0.61, p<0.001$).

**Table 7:** Pearson correlation coefficient between generalization measures and the relative RMSE. $r_{cv}$ denotes the coefficient for leave-one-representation-out cross-validation on random splits and $r_{a}$ the coefficient measured on all aggregated predictions

	All		Embeddings		Fingerprints		Descriptors	
	$r_{cv}$	$r_{a}$	$r_{cv}$	$r_{a}$	$r_{cv}$	$r_{a}$	$r_{cv}$	$r_{a}$
TopoLearn (C)	0.61 ± 0.19	0.54	0.65 ± 0.1	0.41	0.63 ± 0.25	0.62	0.48 ± 0.07	0.35
TopoLearn (PH)	**0.58** ± **0.2**	**0.45**	**0.65** ± **0.12**	**0.51**	**0.62** ± **0.24**	**0.59**	**0.46** ± **0.11**	**0.15**
ROGI-XD	0.26 ± 0.22	0.28	0.14 ± 0.13	0.16	0.45 ± 0.12	0.41	0.16 ± 0.23	0.14
RMODI	− 0.18 ± 0.2	− 0.34	− 0.01 ± 0.14	− 0.13	− 0.39 ± 0.1	− 0.55	− 0.12 ± 0.14	− 0.16
SARI	–	–	–	–	0.36 ± 0.15	0.24	–	–

### Interpretability of topological properties

**Fig. 11:**
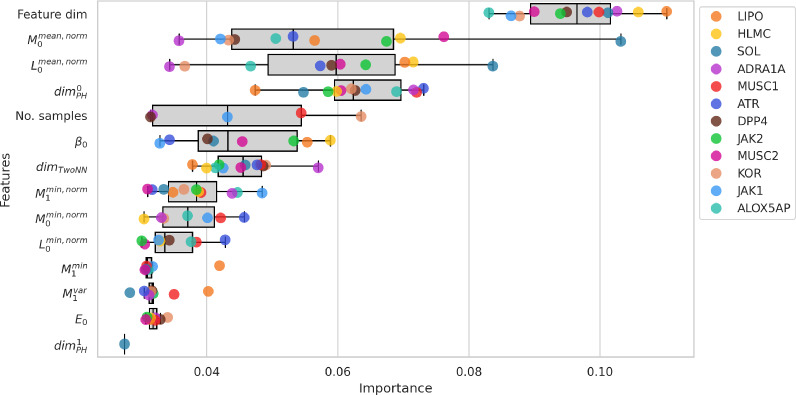
Top 10 feature importances of random forest models for leave-one-dataset-out cross-validation

Having demonstrated that TopoLearn can predict generalization from persistent homology features, we aim to further investigate the model’s learning process and identify the topological configurations that characterize an effective representation. To do so, we aggregate the feature importances of the random forest models trained in the LODO cross-validation process. In Fig. 11 we visualize the 10 most important TopoLearn (C) features across all datasets. We observe that the control variable feature dimensionality is most critical for making accurate predictions. We believe that high dimensionality covers a broader range of relevant features for the different molecular endpoints, thereby leading to high importance values. We also observe this effect when analyzing each representation type separately. Additionally, sample size is among the top features, known to be critical for machine learning models as more samples usually lead to better model performance. Apart from these control variables, we find several persistent homology descriptors in the highest ranking features, most importantly statistical summaries of persistence lifetimes *L*, persistence midlifes *M* and the Betti numbers $\beta _0$. In addition, the dimensionality estimates $dim_{PH}^0$ and $dim_{TwoNN}$ play an essential role for the prediction of generalization.

**Fig. 12:**
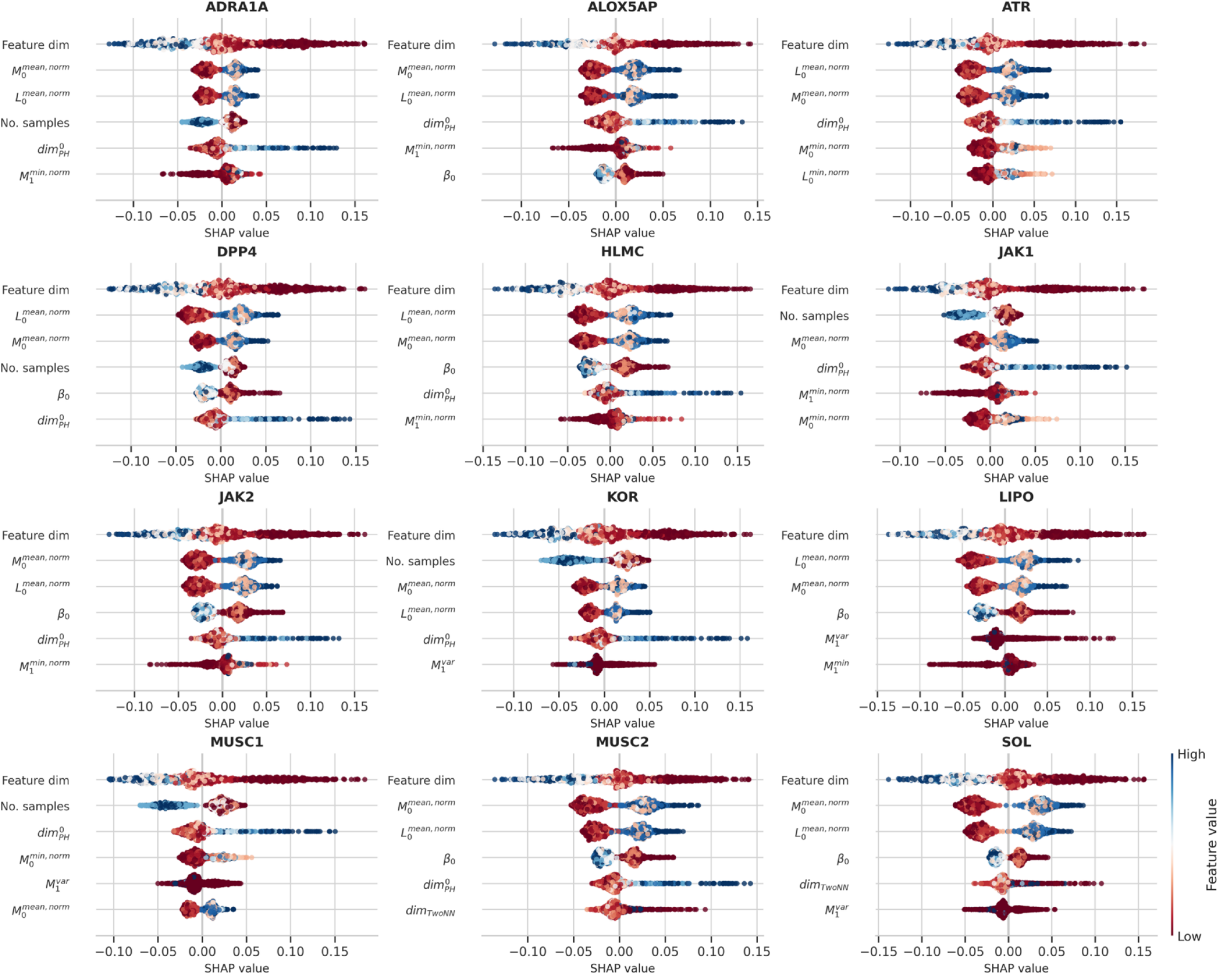
Top 5 SHAP feature effect summaries for LODO cross-validation on randomly split data. The x-axis corresponds to positive or negative effects on the prediction

**Fig. 19:**
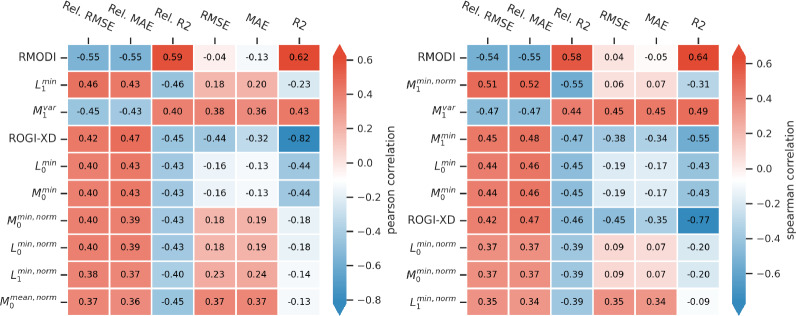
Pearson correlation and Spearman rank correlation coefficient of the most correlated topological features with different error metrics, computed across all randomly-split datasets and binary fingerprint representations

**Fig. 20:**
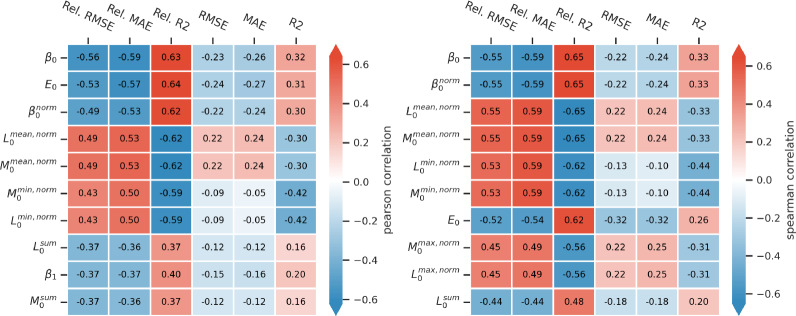
Pearson correlation and Spearman rank correlation coefficient of the most correlated topological features with different error metrics, computed across all randomly-split datasets and embedding representations

**Fig. 21:**
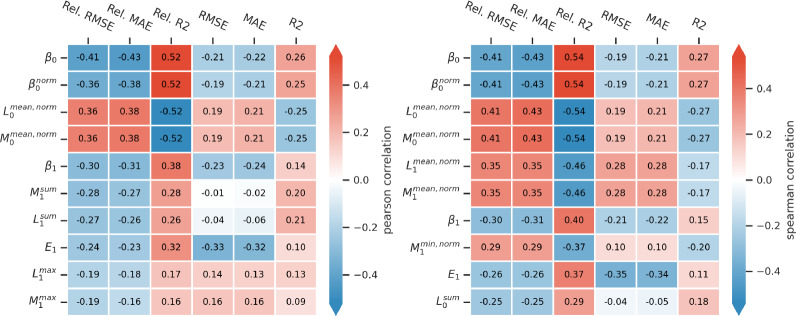
Pearson correlation and Spearman rank correlation coefficient of the most correlated topological features with different error metrics, computed across all randomly-split datasets and molecular descriptor representations

To explore the influence of each variable more thoroughly, we create SHAP summaries for each model, presented in Fig. 12. The figures illustrate that, consistent with intuition, a higher number of features leads to lower generalization errors. Additionally, 0-dimensional Betti numbers are associated with lower model errors. We hypothesize that Betti numbers act as a proxy for sample size, as larger sample sizes result in more persistent features. While long PH feature lifespans are intuitively perceived as important and short lifespans as noise, the feature effect plots reveal that larger interval means are associated with higher model errors. We observe this behavior in both LORO and LODO evaluations, interpreting it as a measure of density; features persist only briefly to form new components in a densely packed feature space. Given the discrete distance metric, binary fingerprints exhibit short feature intervals. We also restrict our analysis to each representation type to confirm that the model does not simply learn to differentiate between representation types, as fingerprints generally outperformed other representations in our experiments. We provide the corresponding SHAP summaries in supplementary file 1. Moreover, we find that the *min* and *mean* aggregation of persistence intervals are consistently more important than other aggregation functions. We also find that $dim^0_{PH}$ plays a significant role in our analysis; as the dimension increases, we observe a corresponding increase in the error, suggesting that higher dimensions are associated with greater errors.

## Discussion

Our evaluation revealed significant correlations with model test errors, indicating that feature spaces exhibit unique topological characteristics closely linked to statistical learning. By fitting TopoLearn to the data, we captured these patterns in a data-driven manner. We derived that simple persistent homology descriptors are the most effective, whereas more complex topological properties are less significant. One limitation of our study is the handling of various distance metrics, as we were unable to find a robust method to normalize persistent homology across them. Another limitation of TopoLearn is that it currently doesn’t consider the labels of a molecular machine learning task. Utilizing TopoLearn as a generalization measure reduces the necessity for exploratory approaches in molecular machine learning, leading to faster identification of effective representations. We believe, that this research direction will inspire several follow-up projects, such as using topological descriptors as a form of density measure of representation spaces or computing topological consistency across representations. Additionally, our results contribute to the design of learned representations and have the potential to act as regularizers. In the spirit of foundation models, we also forsee to expand TopoLearn to a significantly larger collection of molecular datasets and representations. This will cover a greater diversity of topological configurations and dataset types, thereby further enhancing its predictive capabilities. In this context, it would also be interesting to further evaluate the validity of TopoLearn, for example by exploring alternative splitting strategies such as cluster-based splitting. Finally, other summaries of persistent homology, such as persistence landscapes or persistence images, could be an interesting direction for future work in the area of generalization measures.

## Conclusion

Leveraging techniques from topological data analysis, this study addresses the challenge of systematic representation selection in molecular machine learning. Our experiments demonstrated that topological features derived from persistent homology are effective predictors of generalization. The proposed method, TopoLearn, can act as a novel generalization measure by predicting the test error of molecular machine learning representations based on the topological properties of a dataset’s feature space. While topological characteristics cannot fully explain test generalization, we find strong correlations with TopoLearn in two cross-validation scenarios, outperforming all other evaluated modelability measures. Employing interpretability techniques, we conclude that effective representations generally have a higher dimensionality and shorter persistent homology lifetimes, suggesting dense feature spaces with less complex topologies.

## Figures and Tables

**Fig. 13:**
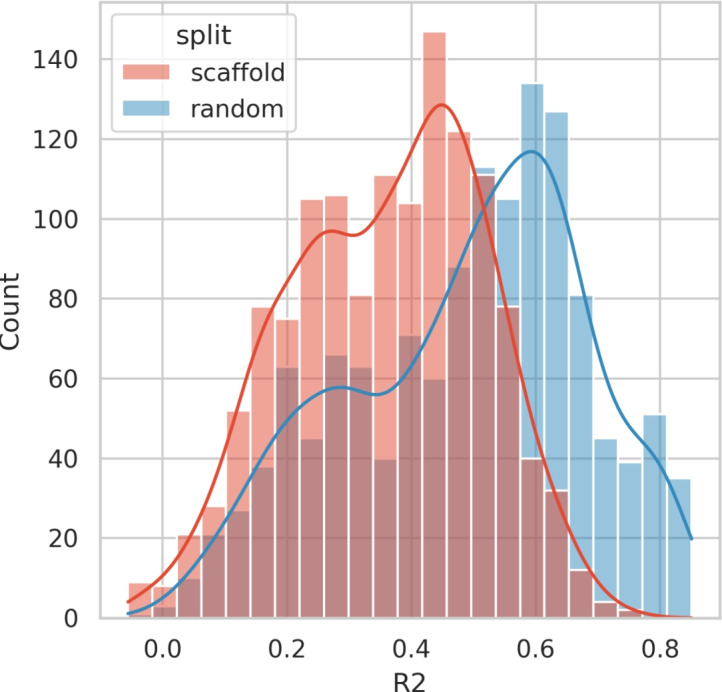
R2 distribution of models trained on scaffold split and random split

**Fig. 14:**
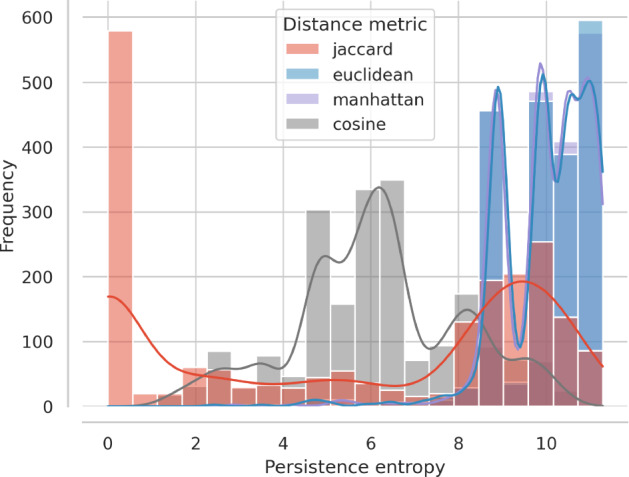
Distribution of persistence entropy computations under different distance metrics

**Fig. 15:**
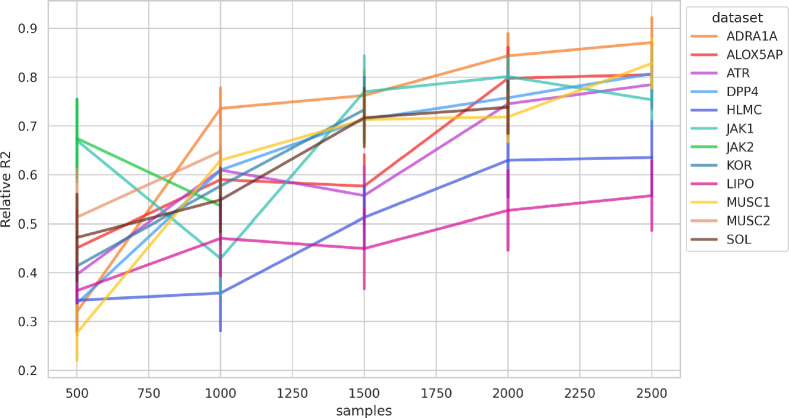
Distribution of normalized $R^2$ across randomly split datasets and sample sizes for different seeds

**Fig. 16:**
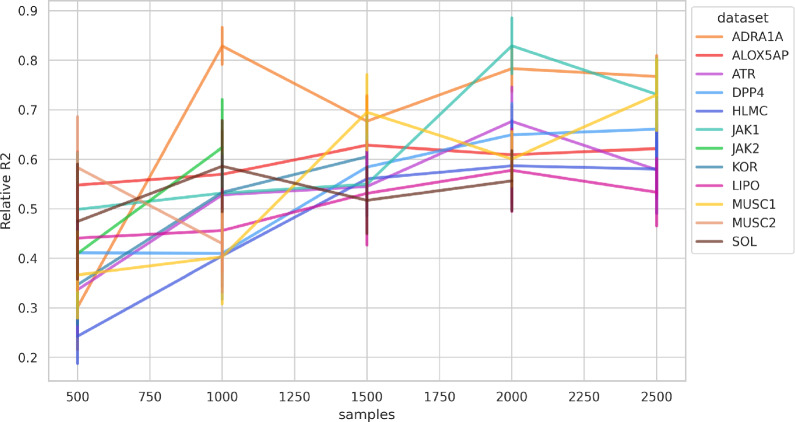
Distribution of normalized $R^2$ across scaffold split datasets and sample sizes for different seeds

**Fig. 17:**
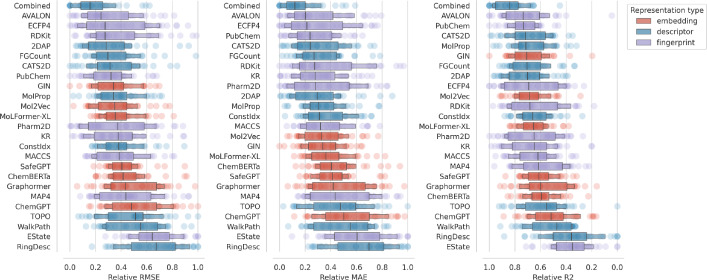
Normalized test error distribution per representation across all datasets for sample sizes $\ge$ 1000 on scaffold-split data

**Fig. 18:**
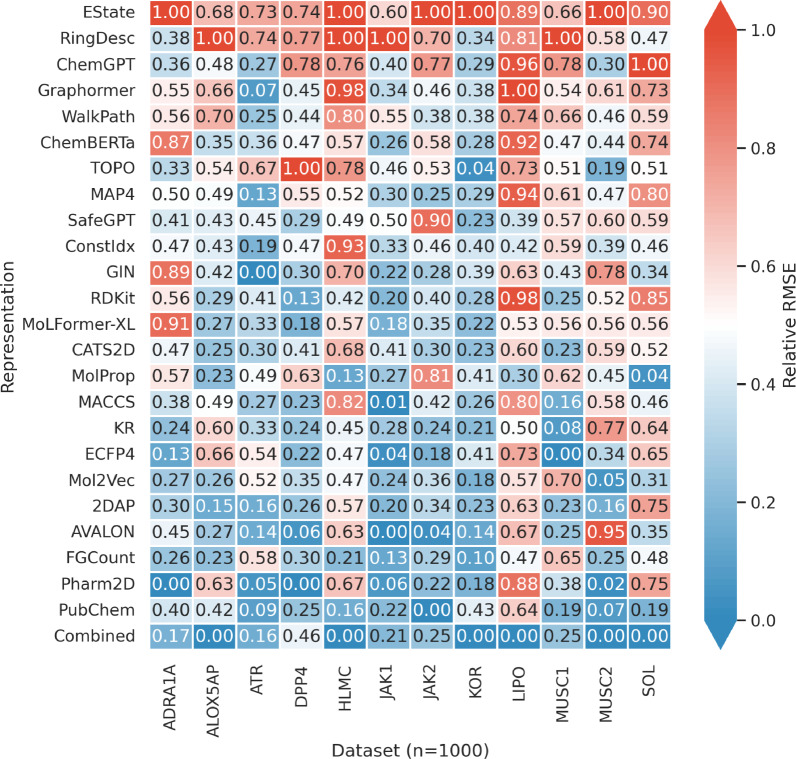
Heatmap of relative test RMSE per dataset and representation for sample size $n=1000$. Blue highlights low errors, where 0 corresponds to the best error achieved on the dataset and red indicates high errors, with 1.0 being the worst possible test error on the dataset

## Data Availability

The datasets supporting the conclusions of this article are compressed as zip files and can be downloaded using git-lfs. The code and the data is publicly available on GitHub: https://github.com/Boehringer-Ingelheim/topolearn. In addition, we provide the final TopoLearn (PH) model under MIT license through PyPi, as described in the GitHub repository.

## Appendix A Model training details

### Cross-validation

Certain numeric molecular representations are more effective with particular machine learning estimators, such as binary fingerprints with tree-based ensemble models. While we could analyze different machine learning algorithms and compare them by model type, this approach might overlook potential synergies between representation and model combinations. Instead, we focus on finding the best possible fit by training both tree-based models and fully-connected neural networks, using the better-performing model to compute the final error. This ensures that we select the most suitable algorithm for each representation. We conduct a 5-fold grid-search cross-validation over the search space specified in Tables 8 and 9. For neural networks we use relu activation and early stopping. Given the number of required training runs, we limit the search spaces to the most essential parameters.

**Table 8:** Hyperparameter search space for random forest

Parameter	Values
n_estimators	[100, 200, 300]
max_depth	[5, 10, 20, 50]
min_samples_leaf	[1, 5, 10]

**Table 9:** Hyperparameter search space for neural network model

Parameter	Values
hidden_layer_sizes	[50, 64, 128, 100, 300]
hidden_layers	[1, 2]
learning_rate_init	[0.001, 0.1, 0.0001]
batch_size	[32, 128]

We choose the model with the best hyperparameters and compute the following error metrics between the true labels *y* and the predicted labels ${\hat{y}}$:

$$\begin{aligned} \text {Mean Absolute Error (MAE)} = \frac{1}{n} \sum _{i=1}^{n} |y_i - {\hat{y}}_i| \\ \text {Root Mean Squared Error (RMSE)} = \sqrt{\frac{1}{n} \sum _{i=1}^{n} (y_i - {\hat{y}}_i)^2} \\ \text {Coefficient of determination } R^2 = 1 - \frac{\sum _{i=1}^{n} (y_i - {\hat{y}}_i)^2}{\sum _{i=1}^{n} (y_i - {\bar{y}})^2} \end{aligned}$$

We run our analyses for the random seeds [2023, 2024, 2025, 2026] and average the results for each error metric. Afterwards, the errors are normalized as described in Eq. 9. This normalization preserves the error distribution within a dataset and enables the comparison of representations. However, we recognize that while our error normalization allows for comparisons across datasets, it also removes information about the spread of the error distribution, which can lead to stretched normalized distributions.

### Dataset splitting

For random splitting, we employ a 80:20 train-test split based on the SMILES strings. For scaffold splitting, we use RDKit to compute Bemis-Murcko scaffolds for each molecule. Based on the resulting scaffolds, we perform a 5-group-fold and calculate the results for each fold. As shown in Fig. 13, the scaffold split test data is more challenging to predict across all experiments.

## Appendix B Evaluation of experimental results

### Influence of distance metric

One key challenge of our study lies in making topological computations comparable across representations. Most distance measures are specifically tailored for a data type, such as the Euclidean distance $d_{\text {E}}(A, B) = \sqrt{(x_2 - x_1)^2 + (y_2 - y_1)^2}$ for continuous and the Jaccard distance $d_J(A, B) = 1 - \frac{|A \cap B|}{|A \cup B|}$ for binary vectors. For our analysis, we additionally consider the cosine distance $d_{\text {cos}}(A, B) = 1 - \frac{A \cdot B}{\Vert A\Vert \Vert B\Vert }$, which is naturally defined for both binary and continuous vectors and also the Manhattan distance $d_{\text {M}}(A, B) = |x_2 - x_1| + |y_2 - y_1|$. Using the natural choice for each representation type introduces new challenges, as the underlying distance metric significantly influences the properties of topological computations. Taking persistence entropy as an example, Fig. 14 illustrates how the Jaccard distance generally leads to lower values and the Euclidean and Manhattan distance reside on the higher end. Along with intuition, cosine distance takes up values in the middle. Normalizing persistence intervals by dividing them by the total sum of persistence is feasible. However, our findings indicate that varying distance measures produce very different features, thereby significantly altering the captured information. As a consequence, all analyses are subject to the selected distance measure. We decide to use the natural choice of Euclidean distance for all continuous vectors and the Jaccard distance for binary vectors and leave the further analysis and unifying of distance measures for future work.

### Influence of sample size

Our experiments reveal a significant connection between sample size and test error. As illustrated in Figs. 15 and 16, larger sample sizes result in higher $R^2$ values, indicating lower errors. Additionally, we observe that the datasets exhibit varying levels of difficulty, a finding that remains consistent across different random seeds used for dataset splitting.

### Prediction error analysis

Figure 17 shows the test error distribution for different representations across all datasets. Similar to the findings from randomly split data, the concatenation of features works well for extrapolation and exhibits a relatively small variance. Additionally, Fig. 18 presents a detailed summary of the normalized test RMSE for a fixed sample size ($n=1000$). This highlights the challenges of each representation for the different datasets and emphasizes the task-dependent nature of the molecular representations analyzed. By investigating the red color gradients in Fig. 18, we identify LIPO and SOL as the most challenging datasets within a scaffold split context, as they consistently result in high errors across numerous representations (see Figs. 19, 20, 21).

### Correlation and feature influence

We find that correlations are generally higher when each representation type is analyzed separately. For binary fingerprints, the modelability indices RMODI ($r=0.62, p<0.001$) and ROGI-XD ($r=-0.82, p<0.001$) show a strong correlation with $R^2$. Furthermore, persistent homology descriptors of homological dimension 1 are more frequently among the most correlated properties for binary fingerprints and molecular descriptors. For embedding representations, persistence entropy exhibits a strong correlation ($r=-0.59, p<0.001$) with the mean absolute error. Similar results for scaffold-split data are provided in supplementary file 1.

### Topological descriptors and dataset partitioning

Given our access to the complete dataset prior to model training, a real-world scenario is to compute the topological descriptors across the entire dataset. However, this might introduce information about the test topology into the evaluation process. To avoid this potential leakage, we also compute the topological descriptors exclusively on the training subset. Our analysis reveals that the topological descriptors computed on the training set are nearly perfectly correlated with those computed on the entire dataset. Consequently, we assume that the intrinsic topological features of the data remain stable, regardless of the specific partitioning used during model training.

### Task-dependence and including label information

Figure 18 demonstrates that the representations are inherently task-dependent, i.e. different target variables lead to different results. This challenges our initial assumption that predictive performance can be inferred solely from topological characteristics. While extending TopoLearn to incorporate label information remains for future research, we present several key insights derived from our experiments. Consider a scenario involving multiple datasets that share the same molecular structures, where each molecule is associated with multiple labels. From a topological perspective, this implies that the data points occupy fixed positions in the representation space (for a given representation), while only their associated labels vary. In such cases, topological features alone are insufficient to reliably predict machine learning performance, because how useful the topology is depends on how the data is labeled. In the extreme case of randomly assigned labels, predictive performance cannot be inferred from the structure of the feature space alone. On the other hand, if the representation itself is randomly distributed within the space, relationships between topology and generalizability aren’t meaningful, regardless of the labeling. We conclude that both the structure of the representation and the alignment with label information are critical for understanding and predicting model performance (see Table 10, 11).

### TopoLearn generalization results

Tables 10 and 11 show the correlation between generalization measures and the error on scaffold-splitdata. We find that TopoLearn outperforms all other measures, specifically on binary fingerprints.

**Table 10:** Pearson correlation coefficient between generalization measures and the relative RMSE

	All		Embeddings		Fingerprints		Descriptors	
	$r_{cv}$	$r_{a}$	$r_{cv}$	$r_{a}$	$r_{cv}$	$r_{a}$	$r_{cv}$	$r_{a}$
TopoLearn (C)	0.53 ± 0.19	0.56	0.44 ± 0.32	0.44	0.60 ± 0.19	0.66	0.55 ± 0.18	0.52
TopoLearn (PH)	**0.47** ± **0.24**	**0.50**	**0.43** ± **0.32**	**0.44**	**0.56** ± **0.23**	**0.63**	**0.42** ± **0.29**	**0.41**
ROGI-XD	0.18 ± 0.18	0.35	0.20 ± 0.42	0.30	0.39 ± 0.25	0.51	0.11 ± 0.19	0.21
RMODI	− 0.24 ± 0.22	− 0.26	− 0.14 ± 0.19	− 0.1	− 0.51 ± 0.22	− 0.49	− 0.14 ± 0.17	− 0.11
SARI	–	–	–	–	− 0.17 ± 0.13	0.32	–	–

$r_{cv}$ denotes the coefficient for leave-one-dataset-out cross-validation on scaffold splits and $r_{a}$ the coefficient measured on all aggregated predictions

**Table 11:** Pearson correlation coefficient between generalization measures and the relative RMSE

	All		Embeddings		Fingerprints		Descriptors	
	$r_{cv}$	$r_{a}$	$r_{cv}$	$r_{a}$	$r_{cv}$	$r_{a}$	$r_{cv}$	$r_{a}$
TopoLearn (C)	0.51 ± 0.21	0.47	0.44 ± 0.14	0.33	0.65 ± 0.22	0.65	0.37 ± 0.25	0.18
TopoLearn (PH)	**0.49** ± **0.17**	**0.39**	**0.47** ± **0.10**	**0.37**	**0.59** ± **0.21**	**0.62**	**0.38** ± **0.17**	**0.10**
ROGI-XD	0.36 ± 0.21	0.35	0.30 ± 0.11	0.30	0.53 ± 0.11	0.51	0.23 ± 0.19	0.21
RMODI	− 0.15 ± 0.20	− 0.26	− 0.01 ± 0.18	− 0.1	− 0.36 ± 0.15	− 0.49	− 0.05 ± 0.15	− 0.11
SARI	–	–	–	–	0.41± 0.10	0.32	–	–

$r_{cv}$ denotes the coefficient for leave-one-representation-out cross-validation on scaffold splits and $r_{a}$ the coefficient measured on all aggregated predictions

## Additional Information

Additional file 1. Figures for molecular dataset statistics, persistent homology, correlation summaries and SHAP plots.

## Acronyms

ACs: Activity cliffs
ADME: Absorption, distribution, metabolism, and excretion
CLMs: Chemical language models
ECFP: Extended-connectivity fingerprints
GNNs: Graph neural networks
ID: Intrinsic dimension
MACCS: Molecular access system
MAE: Mean absolute error
ML: Machine learning
MODI: Modelability index
MolML: Molecular machine learning
PCA: Principal component analysis
PH: Persistent homology
QSAR: Quantitiative structure-activity relationship
RMODI: Regression modelability index
RMSE: Root mean squared error
ROGI: Roughness index
ROGI-XD: Roughness index extended
SALI: Structure-activity landscape index
SARI: Structure-activity relationship index
SELFIES: Self-referencing embedded strings
SMILES: Simplified molecular input line entry system
TDA: Topological data analysis
