# Supplementary material for: The topology of molecular representations and its influence on machine learning performance
Source: J Cheminform. 2025 Jul 21;17:109. doi: 10.1186/s13321-025-01045-w (PMC12281805; doi:10.1186/s13321-025-01045-w)
Supplement: Supplementary file 1 — Additional file 1. Figures for molecular dataset statistics, persistent homology, correlation summaries and SHAP plots. [file 13321_2025_1045_MOESM1_ESM.pdf]

Supplementary File 1

The Topology of Molecular Representations and Its Influence on Machine Learning Performance

Florian Rottach [1,3], Sebastian Schieferdecker [2], Carsten Eickhoff [3]

Affiliations:

[1] Central Data Science, Boehringer Ingelheim GmbH, Biberach/Riss, Germany

[2] Computational Toxicology, Boehringer Ingelheim Pharma GmbH & Co. KG, Biberach/Riss, Germany

[3] School of Medicine, University of Tübingen, Germany

Table of Contents

Dataset distribution 3

Dataset statistics 7

Persistence diagrams 8

Persistence lifetime distribution 11

Correlation tables for scaffold-split data 14

SHAP summaries 16

Figures

Figure S1: LIPO dataset pairwise ECFP4 Tanimoto distance (left) and clustering of reduced graph Bemis-Murcko scaffolds (right) 3

Figure S2: ADRA1A dataset pairwise ECFP4 Tanimoto distance (left) and clustering of reduced graph Bemis-Murcko scaffolds (right) 3

Figure S3: MUSC1 dataset pairwise ECFP4 Tanimoto distance (left) and clustering of reduced graph Bemis-Murcko scaffolds (right) 3

Figure S4: JAK1 dataset pairwise ECFP4 Tanimoto distance (left) and clustering of reduced graph Bemis-Murcko scaffolds (right) 4

Figure S5: ATR dataset pairwise ECFP4 Tanimoto distance (left) and clustering of reduced graph Bemis-Murcko scaffolds (right) 4

Figure S6: ATR dataset pairwise ECFP4 Tanimoto distance (left) and clustering of reduced graph Bemis-Murcko scaffolds (right) 4

Figure S 7: JAK2 dataset pairwise ECFP4 Tanimoto distance (left) and clustering of reduced graph Bemis-Murcko scaffolds (right) 5

Figure S8: SOL dataset pairwise ECFP4 Tanimoto distance (left) and clustering of reduced graph Bemis-Murcko scaffolds (right) 5

Figure S9: MUSC2 dataset pairwise ECFP4 Tanimoto distance (left) and clustering of reduced graph Bemis-Murcko scaffolds (right) 5

Figure S10: HLMC dataset pairwise ECFP4 Tanimoto distance (left) and clustering of reduced graph Bemis-Murcko scaffolds (right) 6
Figure S11: KOR dataset pairwise ECFP4 Tanimoto distance (left) and clustering of reduced graph Bemis-Murcko scaffolds (right) 6
Figure S12: KOR dataset pairwise ECFP4 Tanimoto distance (left) and clustering of reduced graph Bemis-Murcko scaffolds (right) 6
Figure S13: Dataset descriptors for rotatable bond fraction, fraction of sp3-carbons, TPSA, QED, clogp and heavy atom count. 7
Figure S14: Persistence diagrams for different representations and datasets. 8
Figure S15: Persistence diagrams for different representations and datasets. 9
Figure S16: Persistence diagrams for different representations and datasets. 10
Figure S17: Persistence lifetime distributions of homological dimension 0 for different representations and datasets. 11
Figure S18: Persistence lifetime distributions of homological dimension 0 for different representations and datasets. 12
Figure S19: Persistence lifetime distributions of homological dimension 0 for different representations and datasets. 13
Figure S20: Pearson and Spearman correlation between different topological metrics and test errors across all data........ 14
Figure S21: Pearson and Spearman correlation between different topological metrics and test errors across all embedding representations. 14
Figure S22: Pearson and Spearman correlation between different topological metrics and test errors across all fingerprint representations. 14
Figure S23: Pearson and Spearman correlation between different topological metrics and test errors across all descriptor representations. 15
Figure S24: SHAP summary for leave-one-dataset-out cross-validation models on randomly split data and embedding representations. 16
Figure S25: SHAP summary for leave-one-dataset-out cross-validation models on randomly split data and fingerprint representations. 16
Figure S26: SHAP summary for leave-one-dataset-out cross-validation models on randomly split data and descriptor representations. 17
Figure S27: SHAP summary for leave-one-dataset-out cross-validation models on scaffold split data and all representations. 17
Figure S28: SHAP summary for leave-one-dataset-out cross-validation models on scaffold split data and embedding representations. 18
Figure S29: SHAP summary for leave-one-dataset-out cross-validation models on scaffold split data and fingerprint representations. 18
Figure S30: SHAP summary for leave-one-dataset-out cross-validation models on scaffold split data and fingerprint representations. 19

Dataset distribution

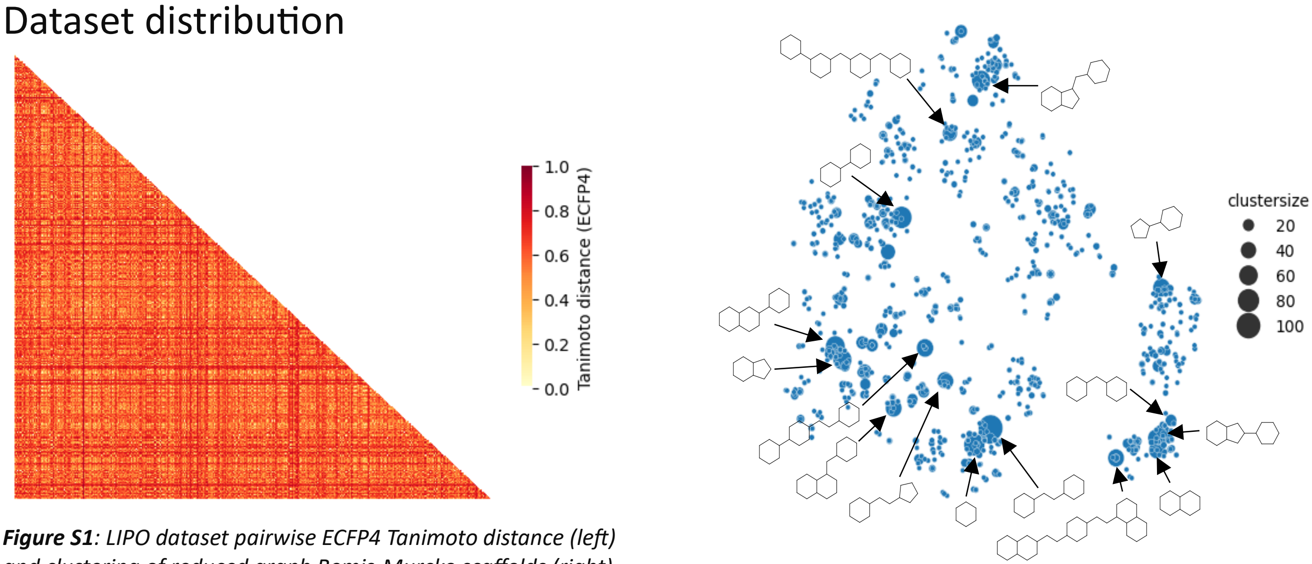

Figure S1*: LIPO dataset pairwise ECFP4 Tanimoto distance (left) and clustering of reduced graph Bemis-Murcko scaffolds (right)*

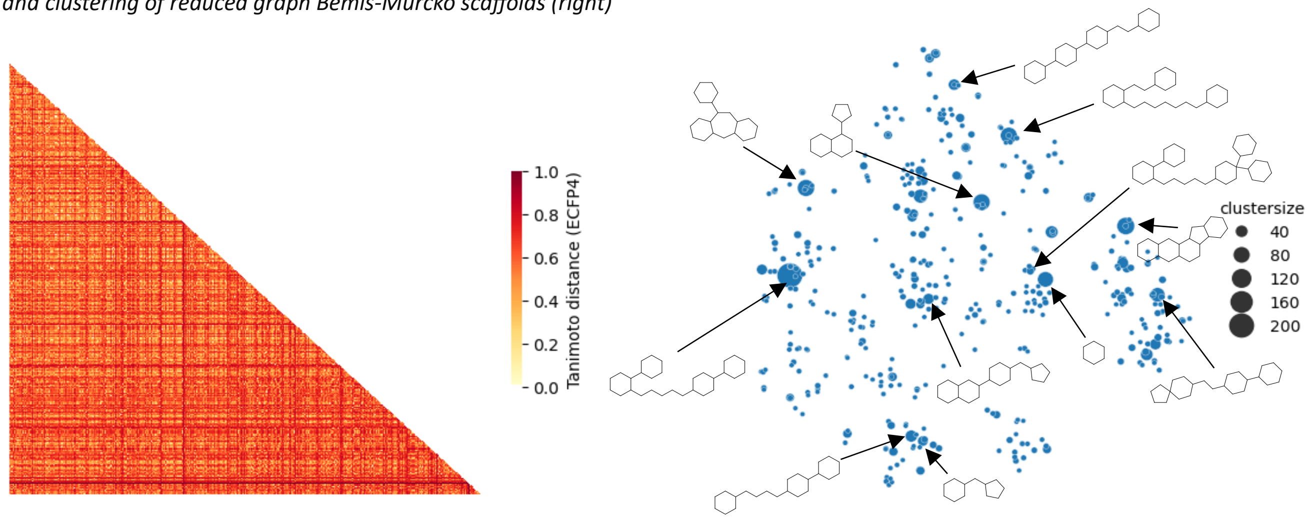

Figure S2*: ADRA1A dataset pairwise ECFP4 Tanimoto distance (left) and clustering of reduced graph Bemis-Murcko scaffolds (right)*

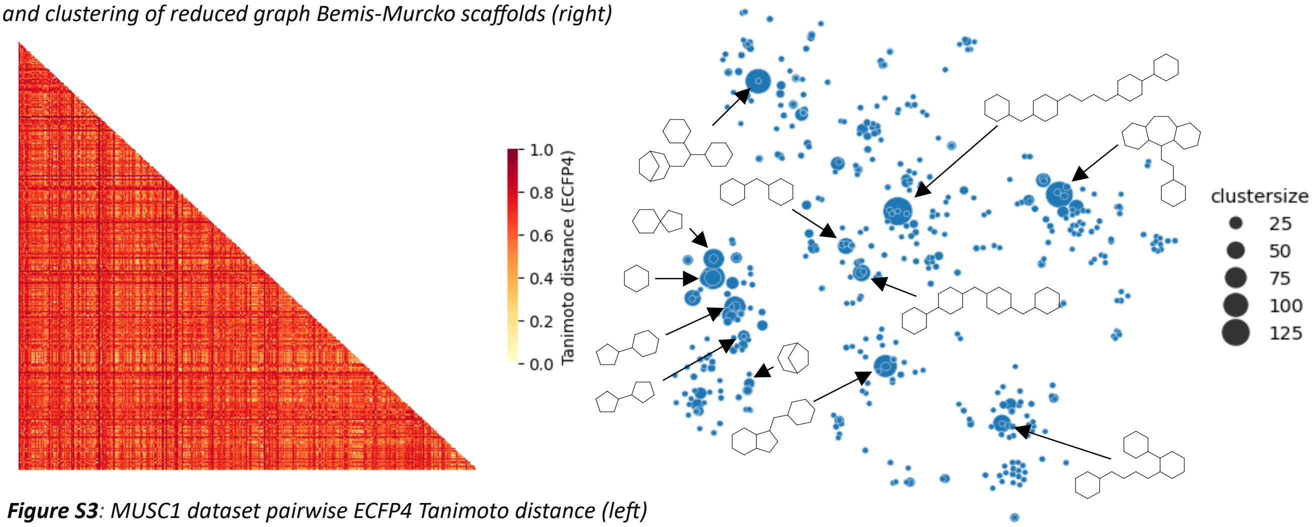

Figure S3*: MUSC1 dataset pairwise ECFP4 Tanimoto distance (left) and clustering of reduced graph Bemis-Murcko scaffolds (right)*

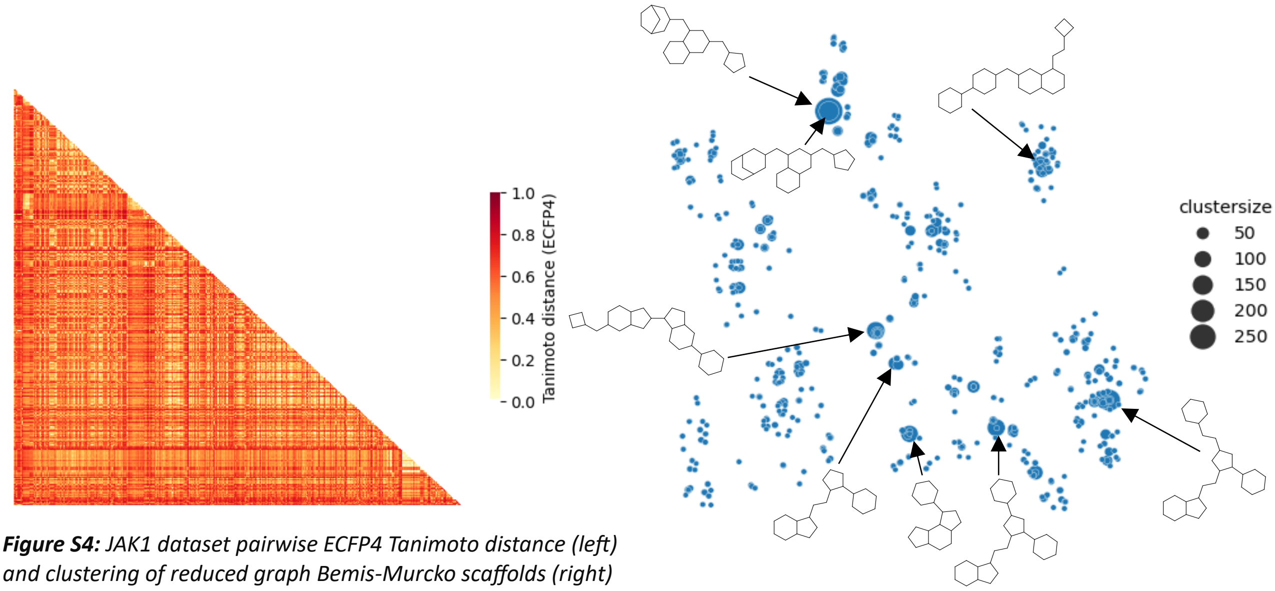

Figure S4: *JAK1 dataset pairwise ECFP4 Tanimoto distance (left) and clustering of reduced graph Bemis-Murcko scaffolds (right)*

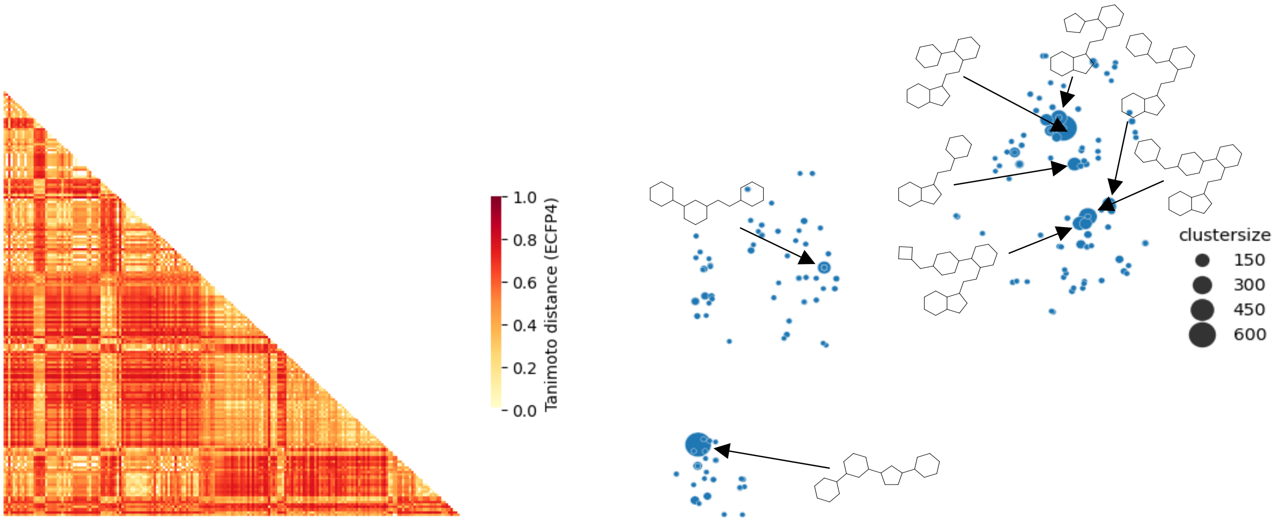

Figure S5: *ATR dataset pairwise ECFP4 Tanimoto distance (left) and clustering of reduced graph Bemis-Murcko scaffolds (right)*

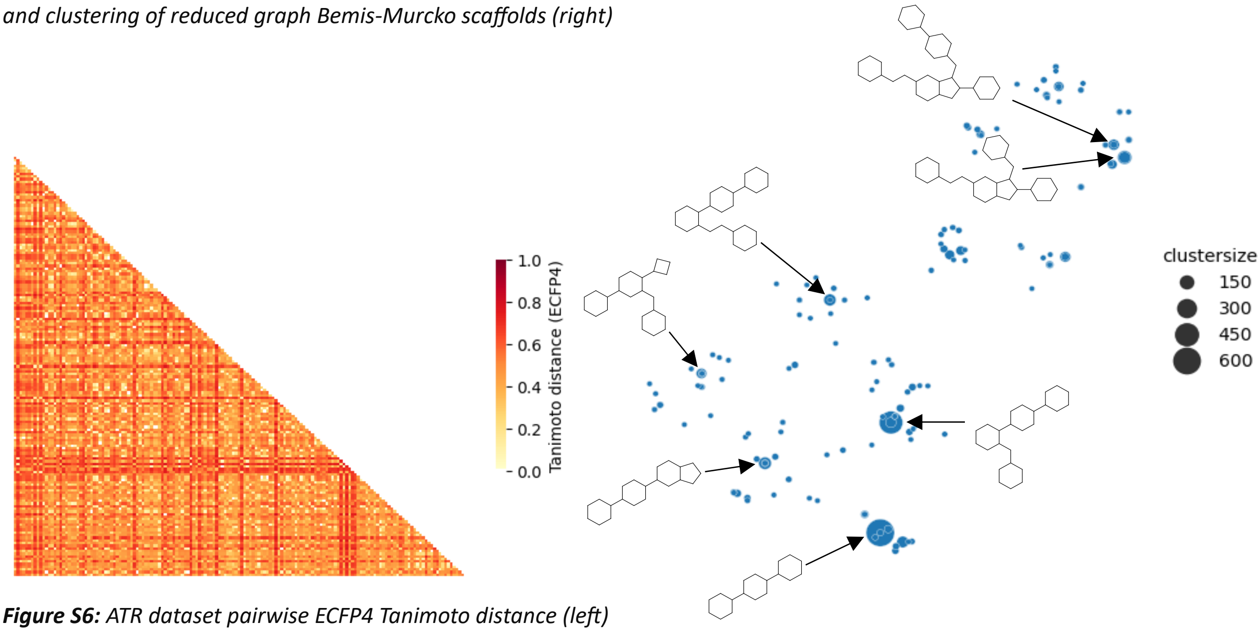

Figure S6: *ATR dataset pairwise ECFP4 Tanimoto distance (left) and clustering of reduced graph Bemis-Murcko scaffolds (right)*

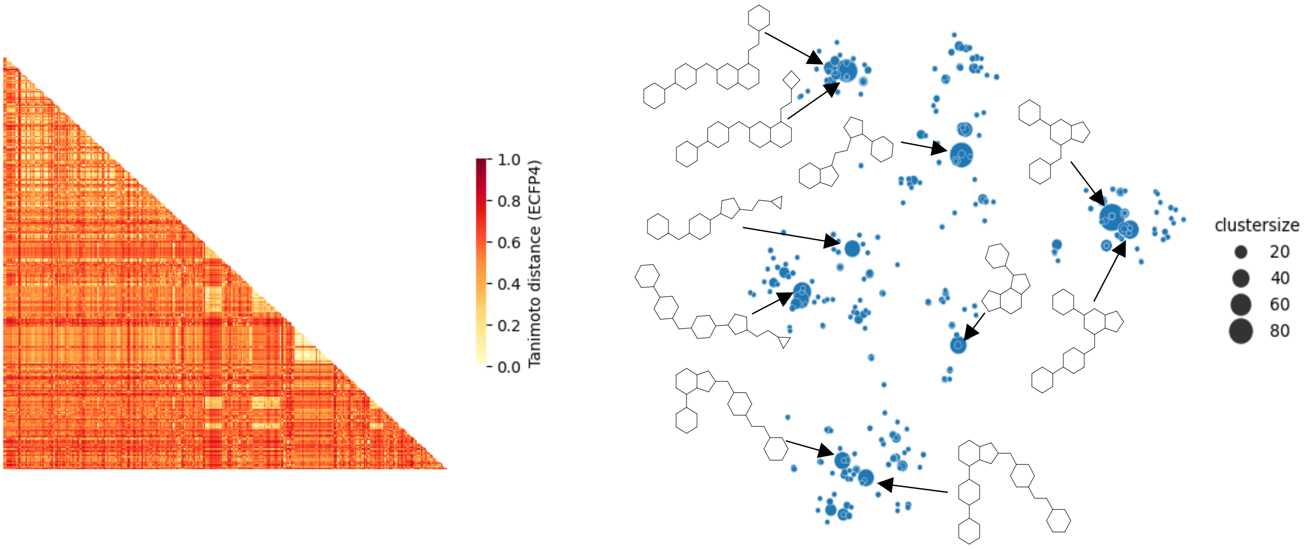

Figure S 7: *JAK2 dataset pairwise ECFP4 Tanimoto distance (left) and clustering of reduced graph Bemis-Murcko scaffolds (right)*

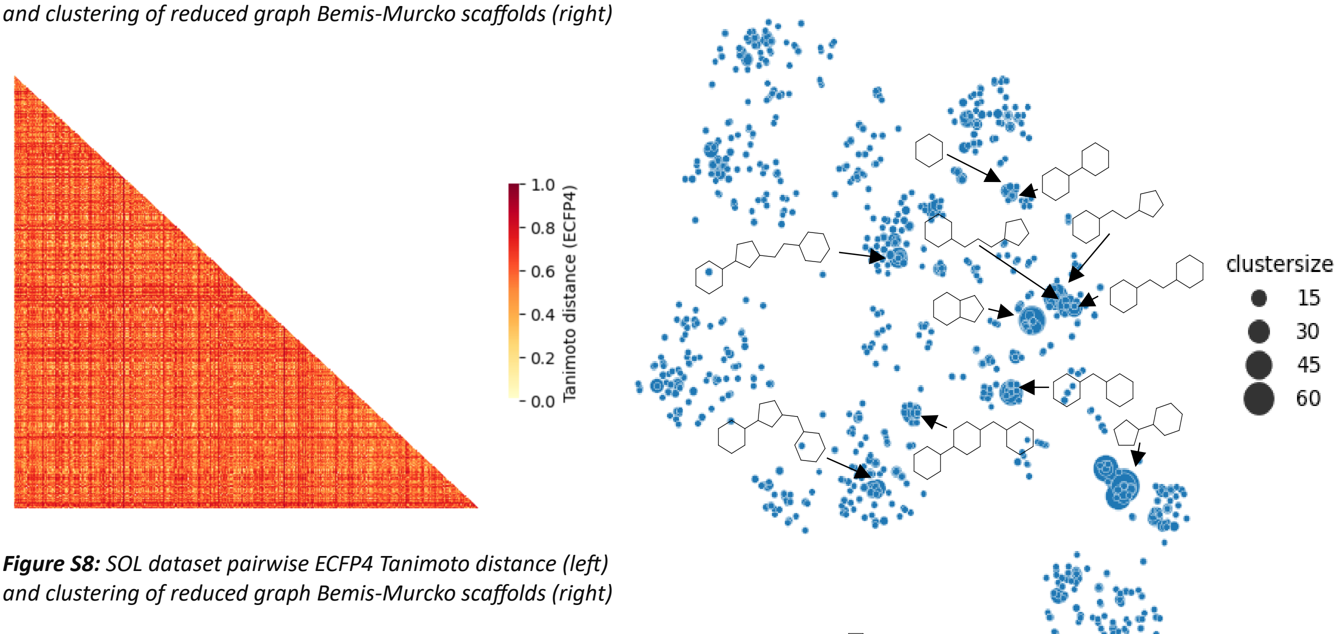

Figure S8: *SOL dataset pairwise ECFP4 Tanimoto distance (left) and clustering of reduced graph Bemis-Murcko scaffolds (right)*

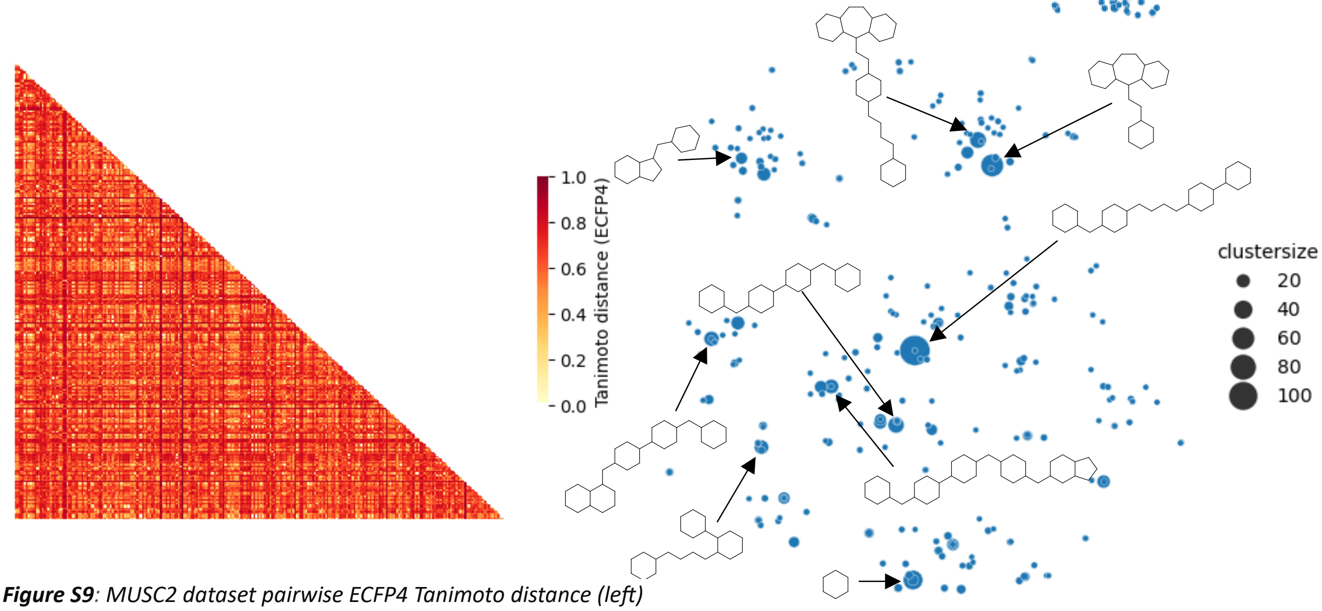

Figure S9*: MUSC2 dataset pairwise ECFP4 Tanimoto distance (left) and clustering of reduced graph Bemis-Murcko scaffolds (right)*

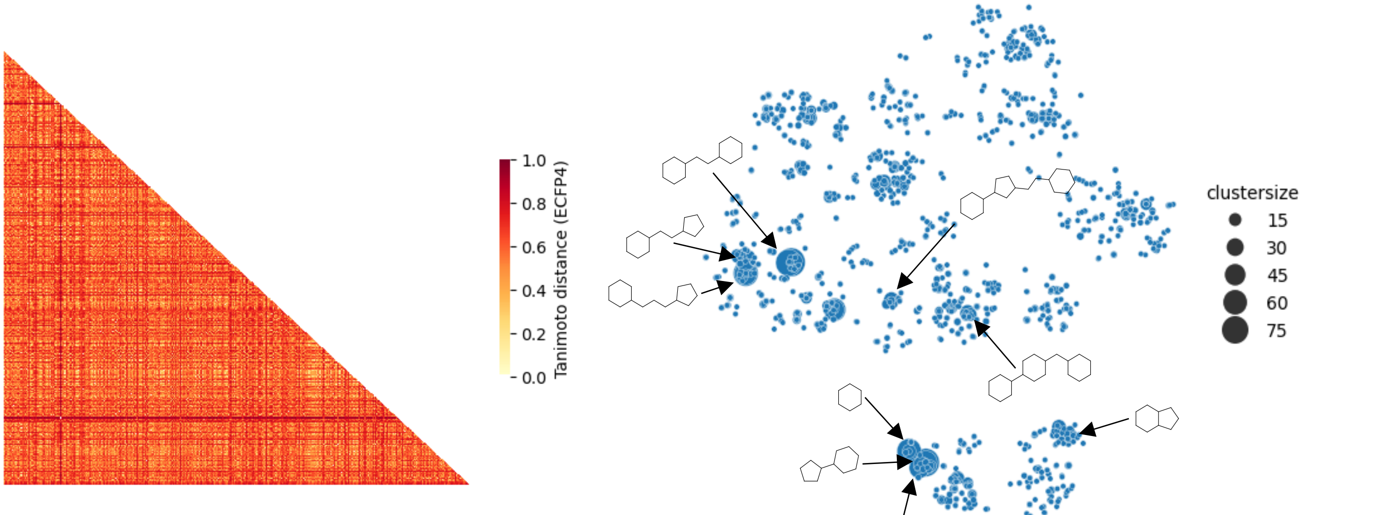

Figure S10: *HLMC dataset pairwise ECFP4 Tanimoto distance (left) and clustering of reduced graph Bemis-Murcko scaffolds (right)*

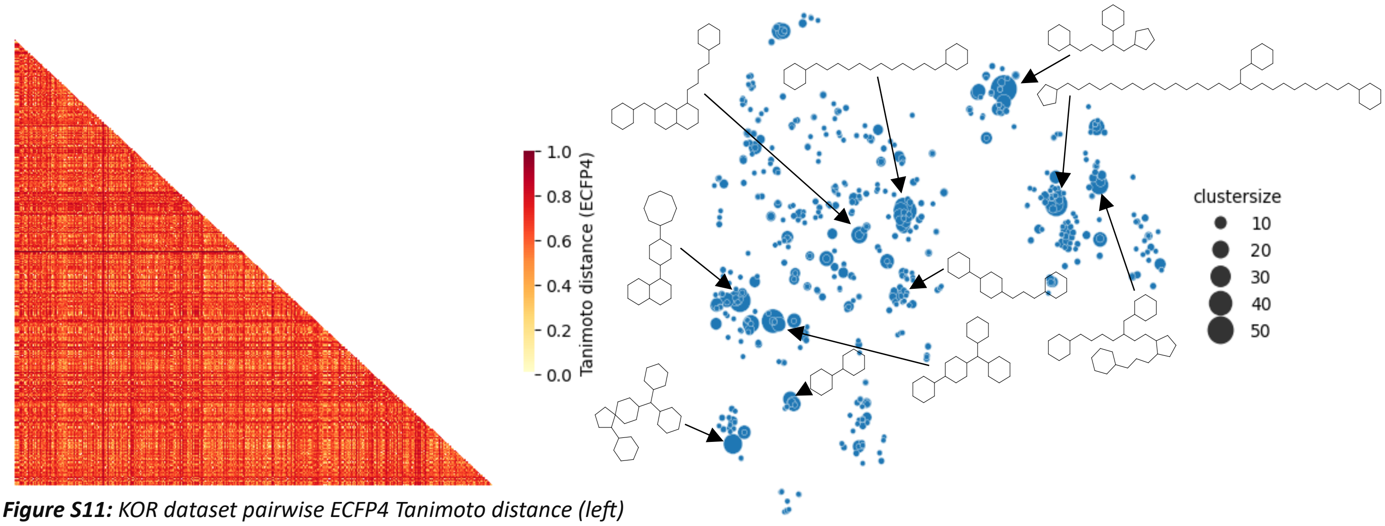

Figure S11: *KOR dataset pairwise ECFP4 Tanimoto distance (left) and clustering of reduced graph Bemis-Murcko scaffolds (right)*

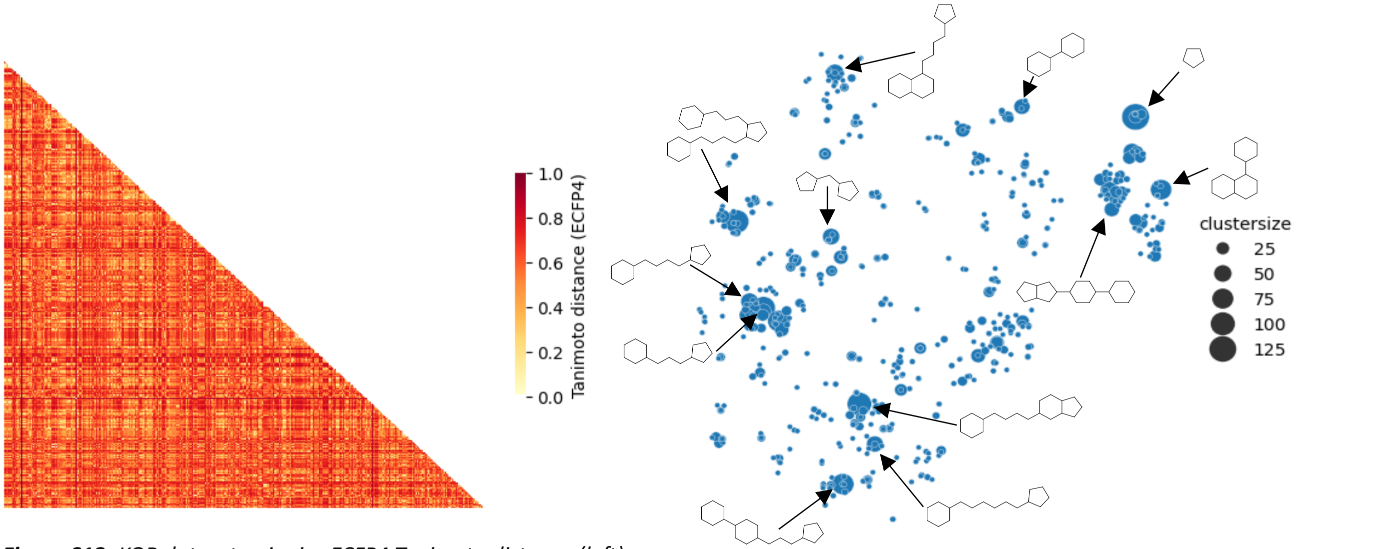

Figure S12: *KOR dataset pairwise ECFP4 Tanimoto distance (left) and clustering of reduced graph Bemis-Murcko scaffolds (right)*

Dataset statistics

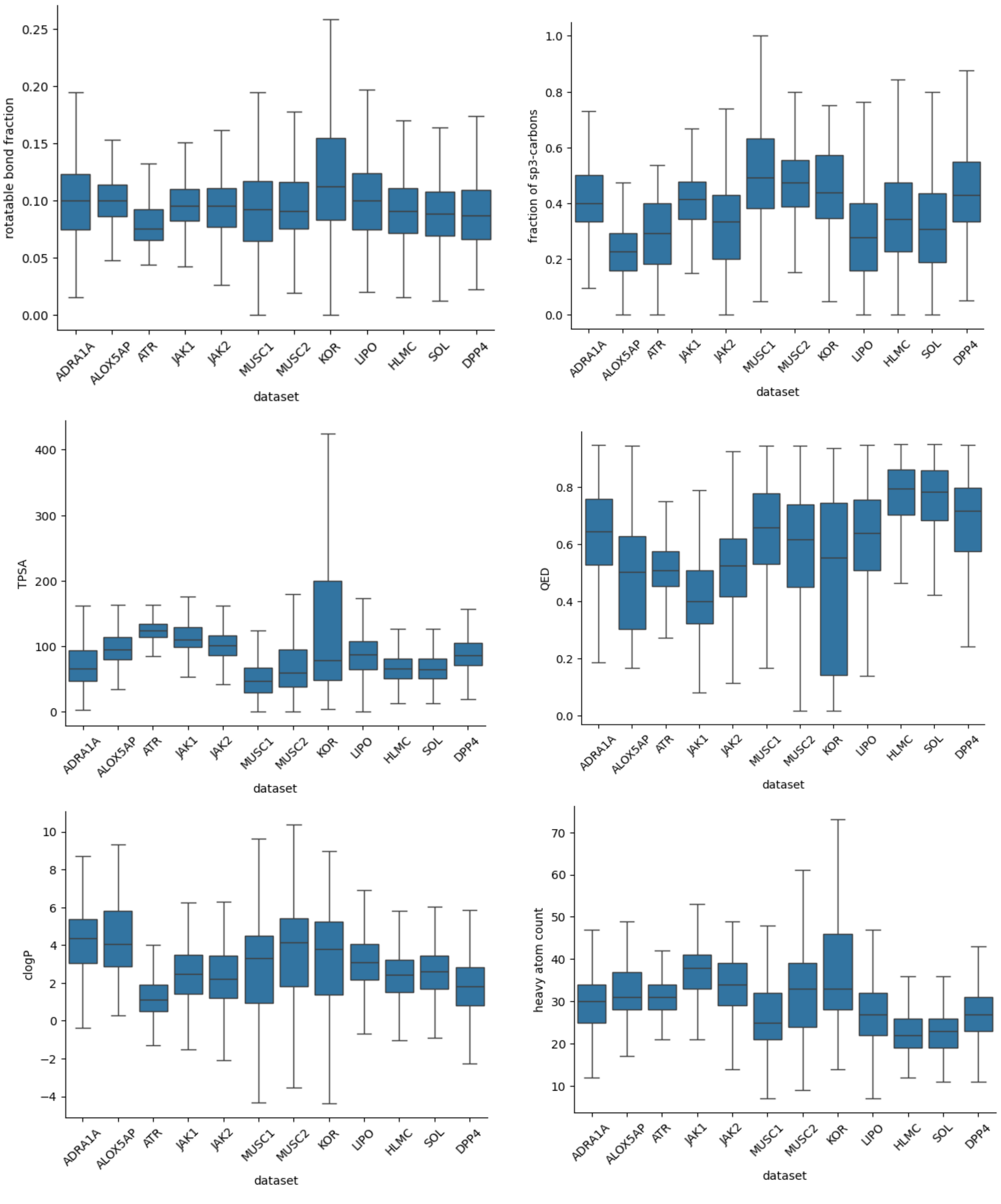

Figure S13: *Dataset descriptors for rotatable bond fraction, fraction of sp3-carbons, TPSA, QED, clogp and heavy atom count.*

Persistence diagrams

For combinatorial reasons, we only provide selected diagrams for sample size 1000 and randomly split data. Other visualizations can be created using the code and data provided at [Boehringer-Ingelheim/topolearn: The topology of molecular machine learning representations].

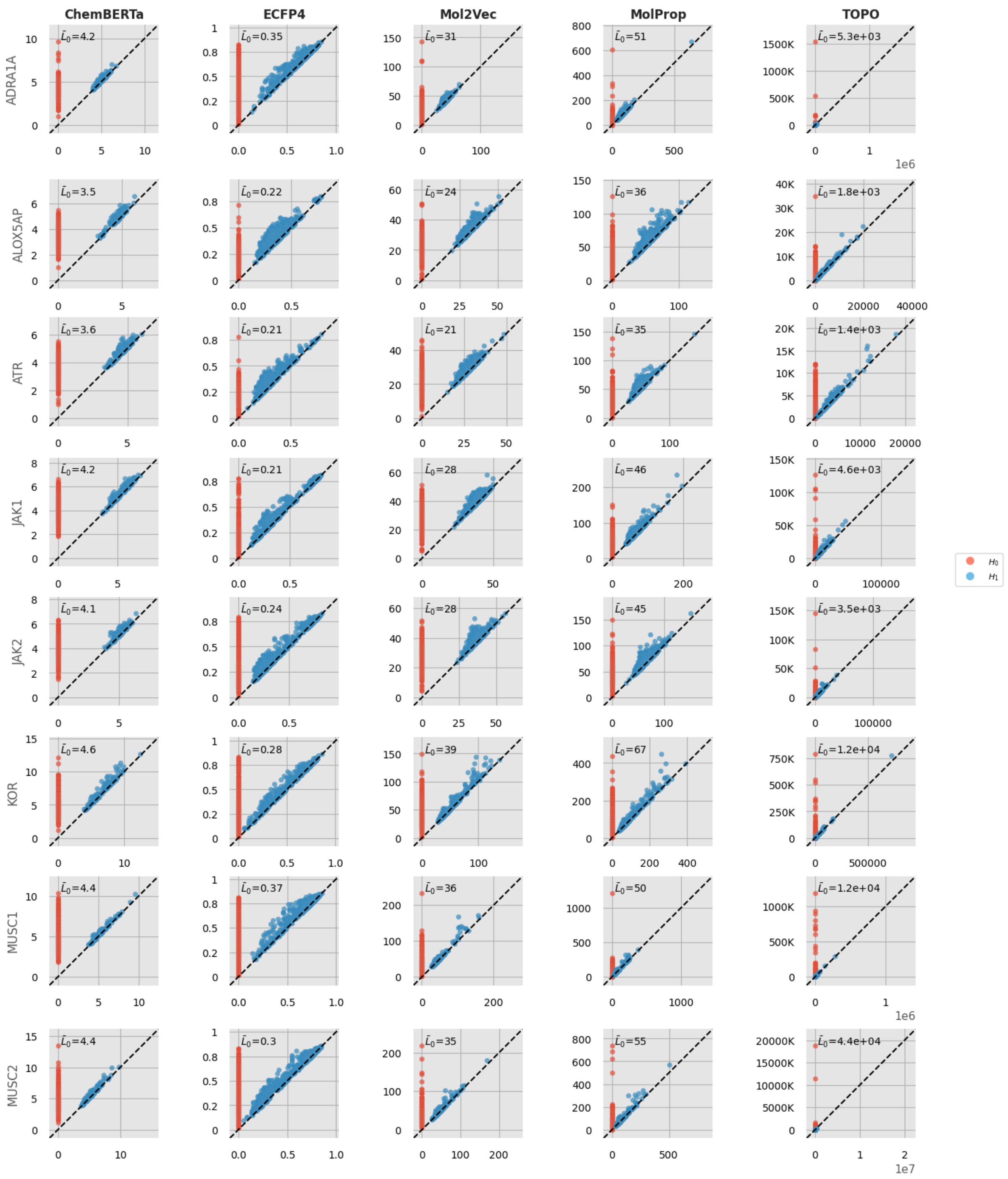

Figure S14: *Persistence diagrams for different representations and datasets.*

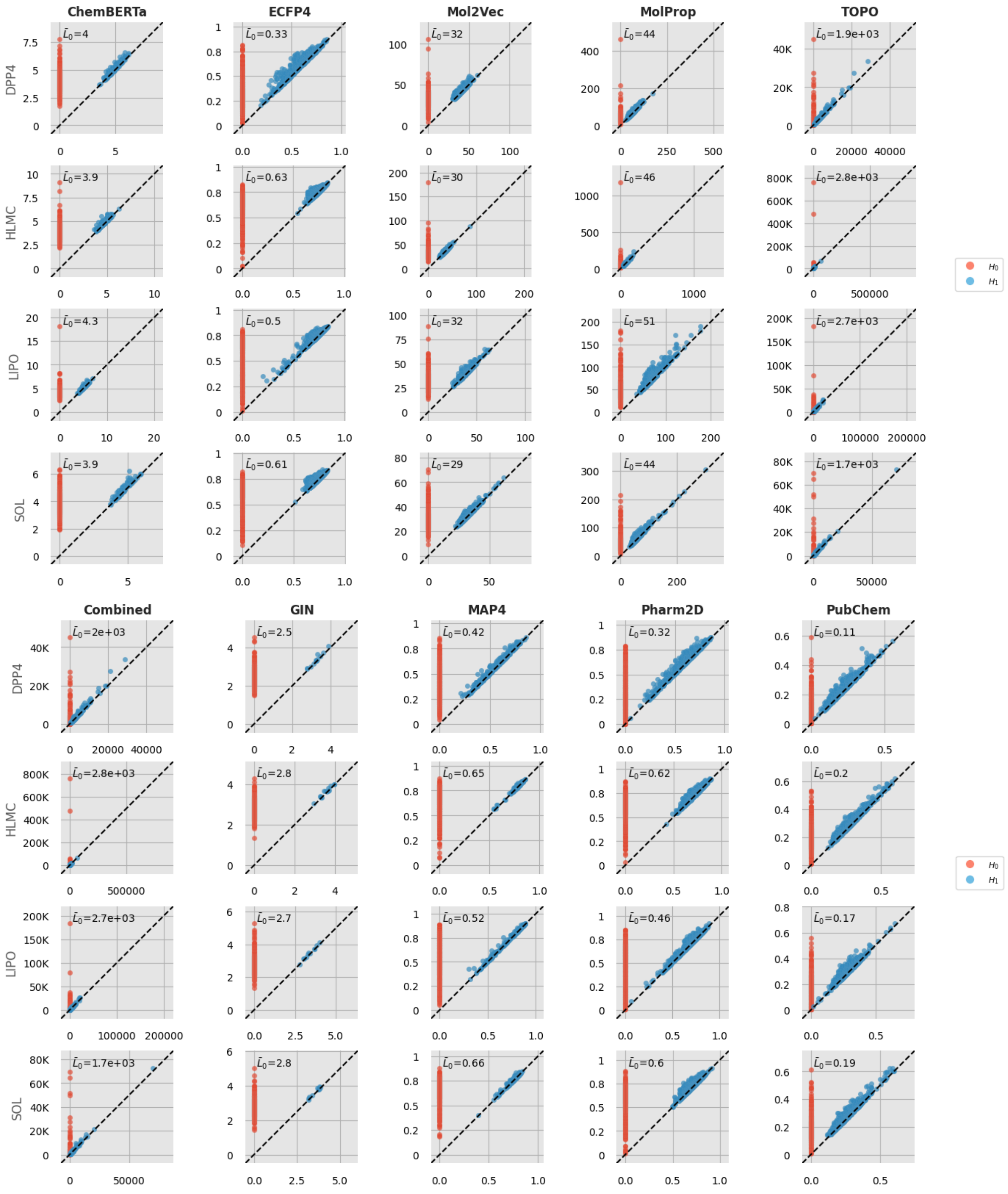

***Figure S15**: Persistence diagrams for different representations and datasets.*

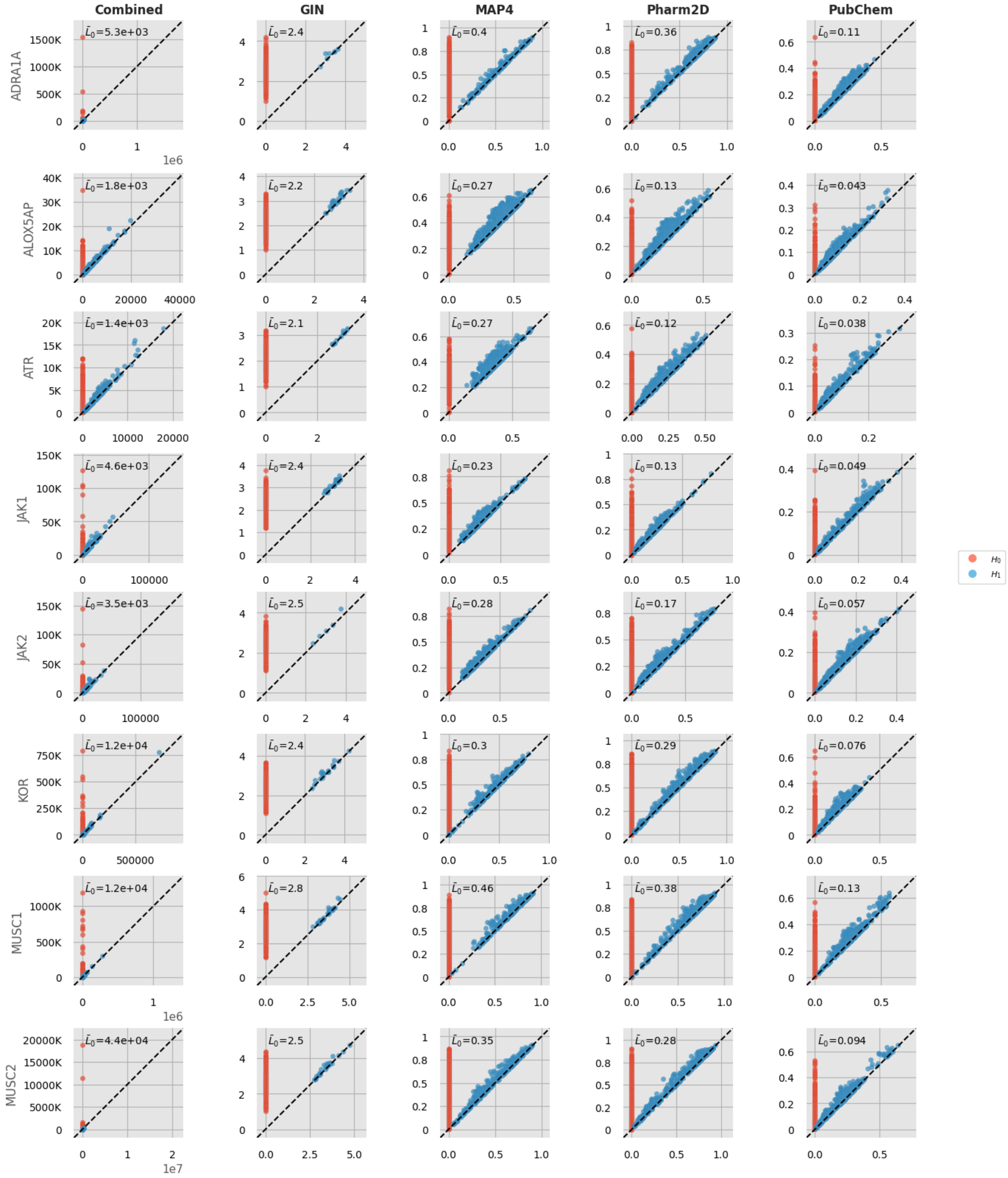

Figure S16: *Persistence diagrams for different representations and datasets.*

Persistence lifetime distribution

For combinatorial reasons, we only provide selected distributions for sample size 1000 and randomly split data. Other visualizations can be created using the code and data provided at [Boehringer-Ingelheim/topolearn: The topology of molecular machine learning representations](Boehringer-Ingelheim/topolearn).

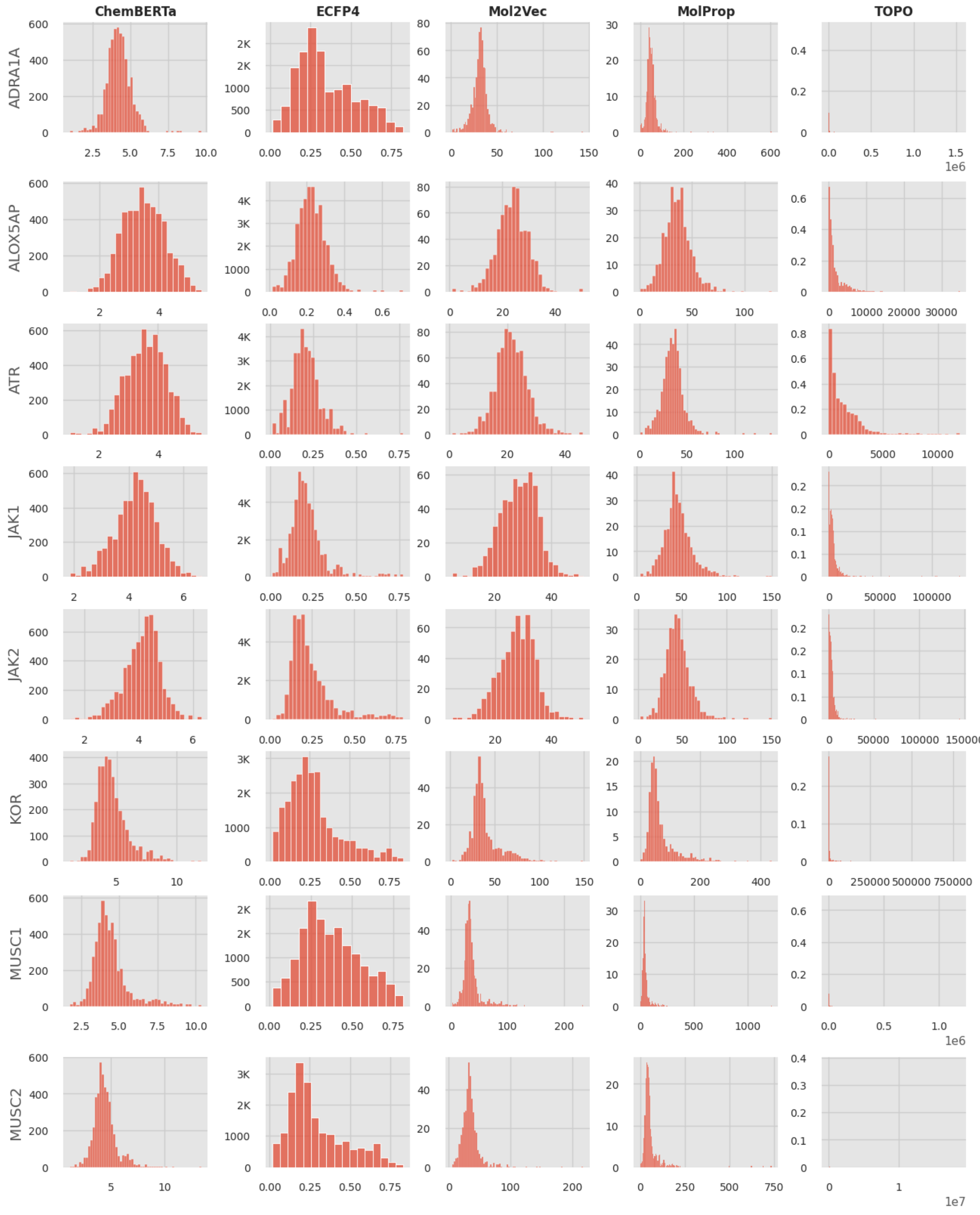

Figure S17: *Persistence lifetime distributions of homological dimension 0 for different representations and datasets.*

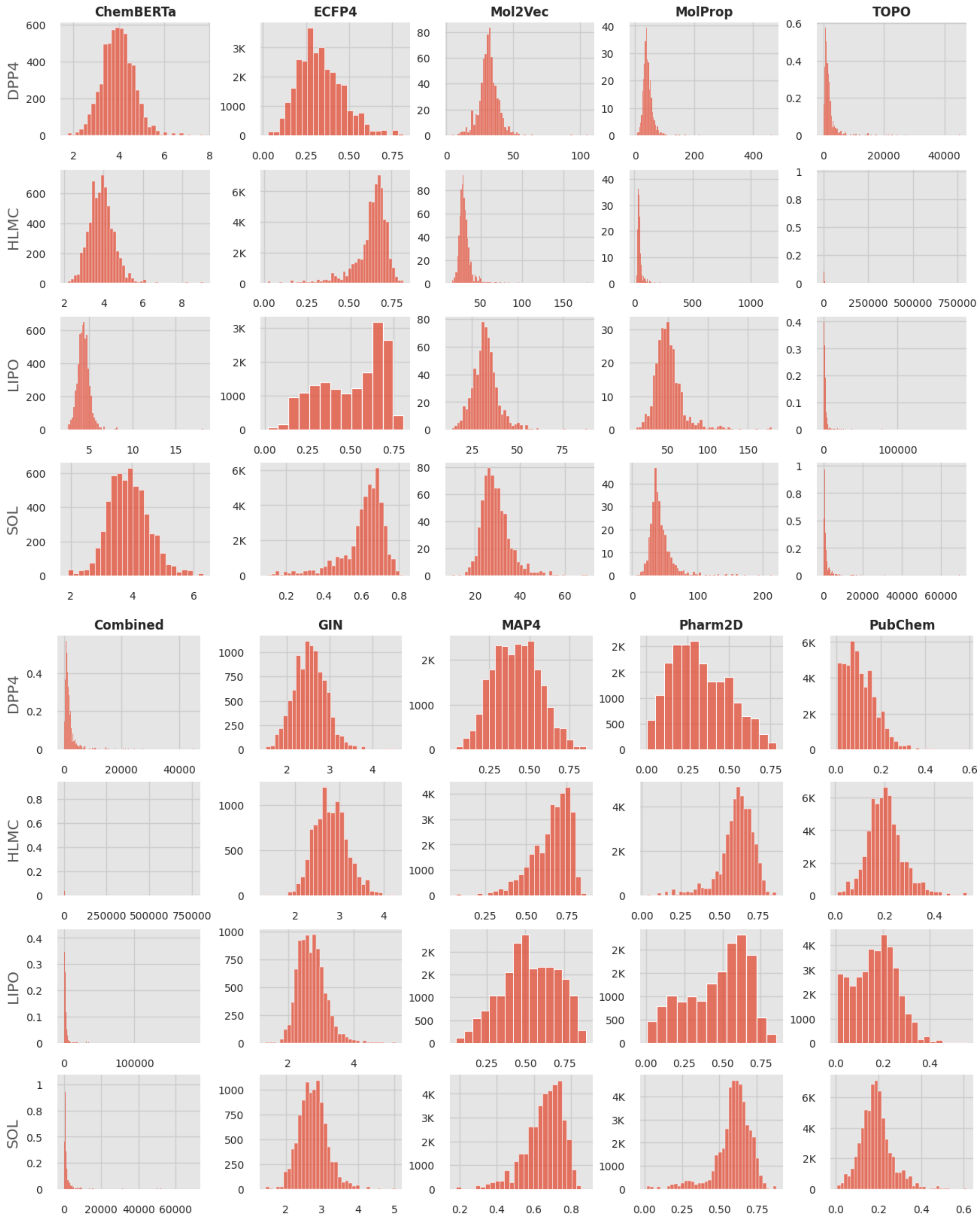

Figure S18: *Persistence lifetime distributions of homological dimension 0 for different representations and datasets.*

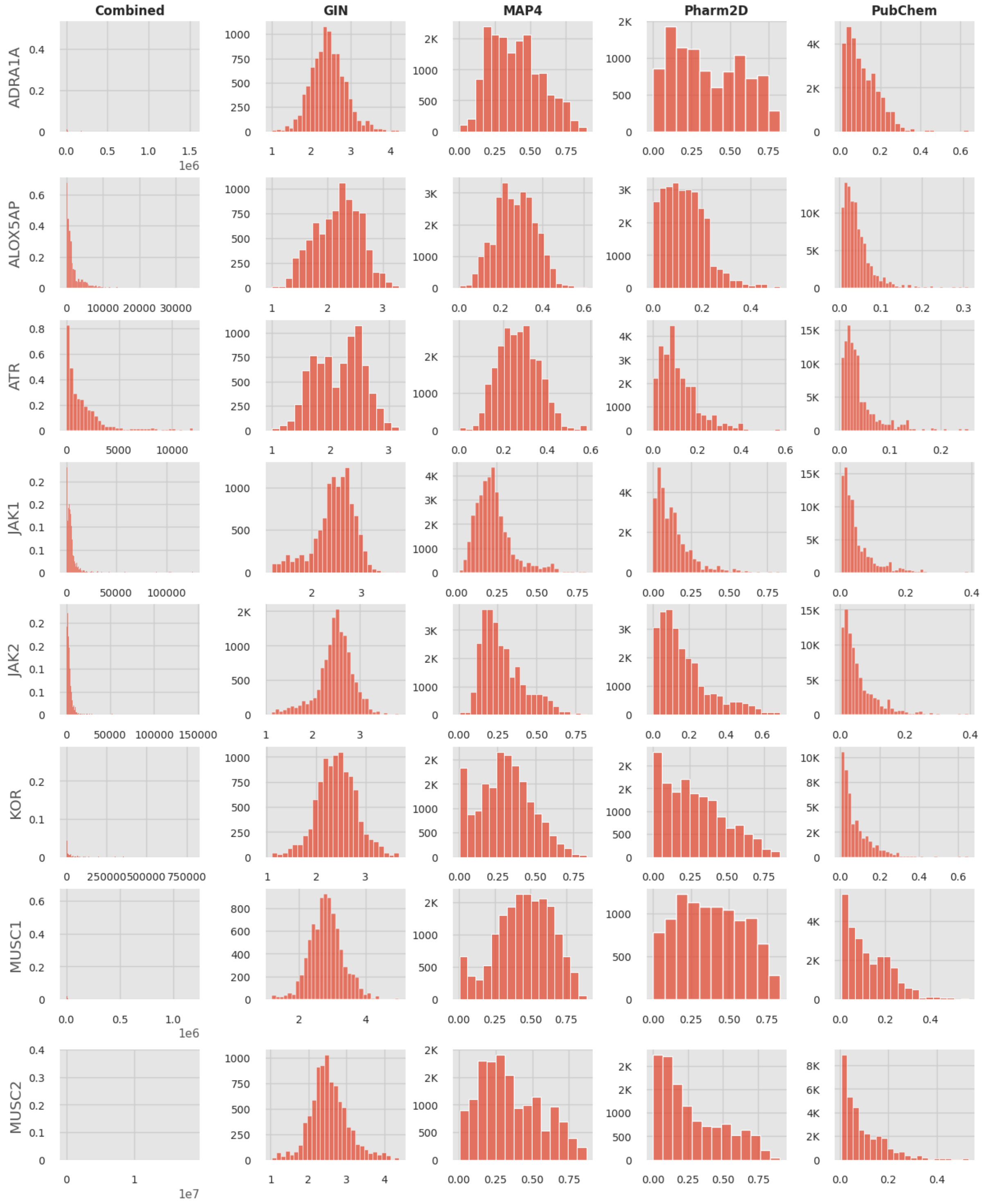

Figure S19: *Persistence lifetime distributions of homological dimension 0 for different representations and datasets.*

Correlation tables for scaffold-split data

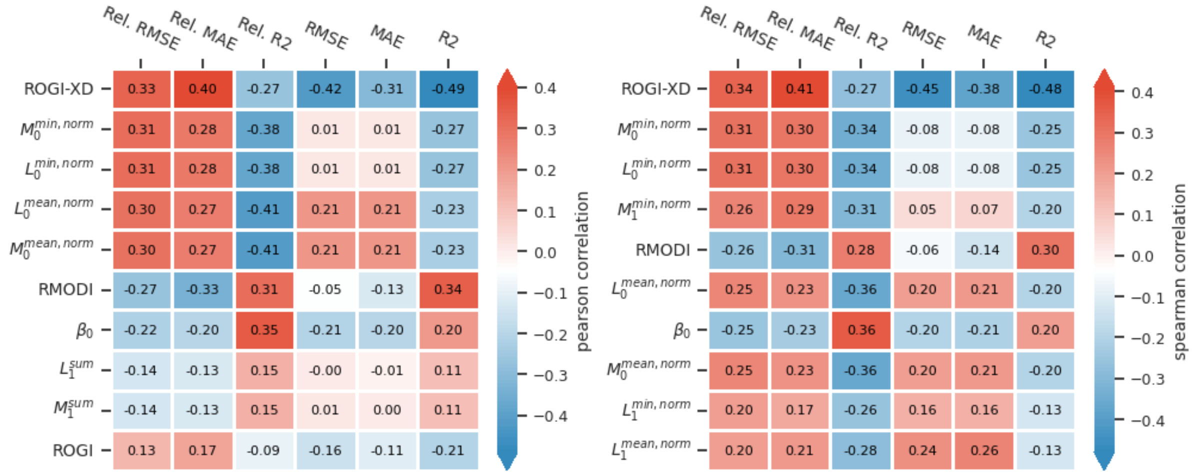

Figure S20: *Pearson and Spearman correlation between different topological metrics and test errors across all data.*

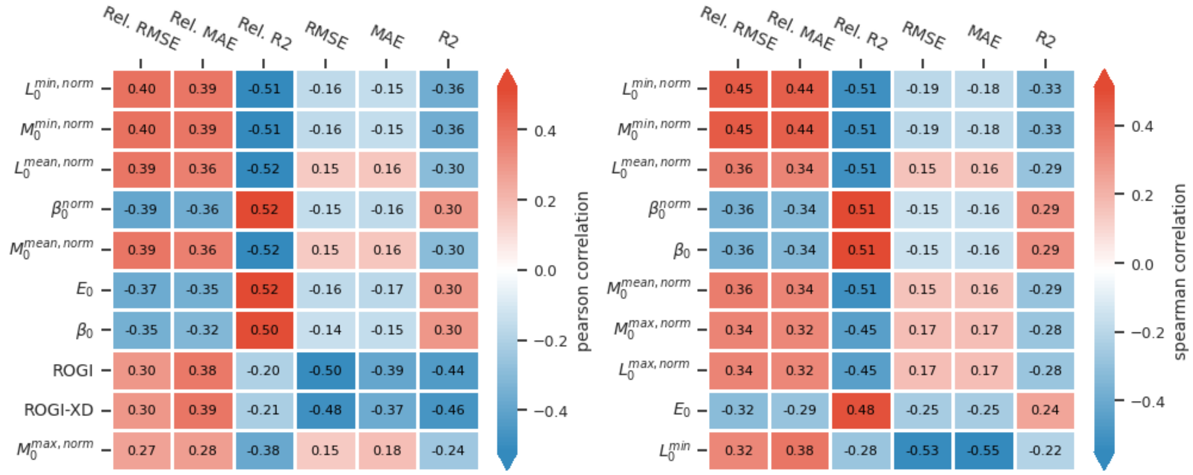

Figure S21: *Pearson and Spearman correlation between different topological metrics and test errors across all embedding representations.*

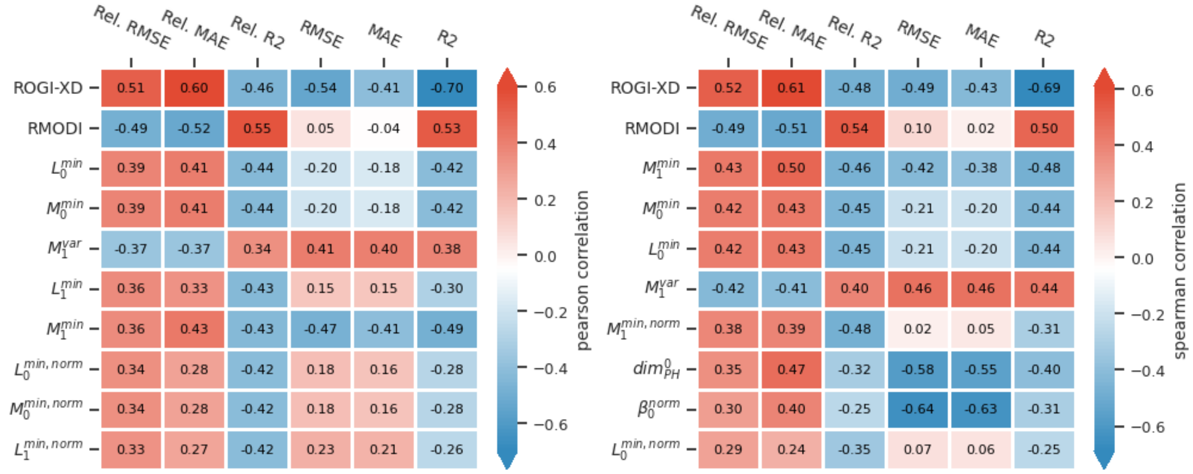

Figure S22: *Pearson and Spearman correlation between different topological metrics and test errors across all fingerprint representations.*

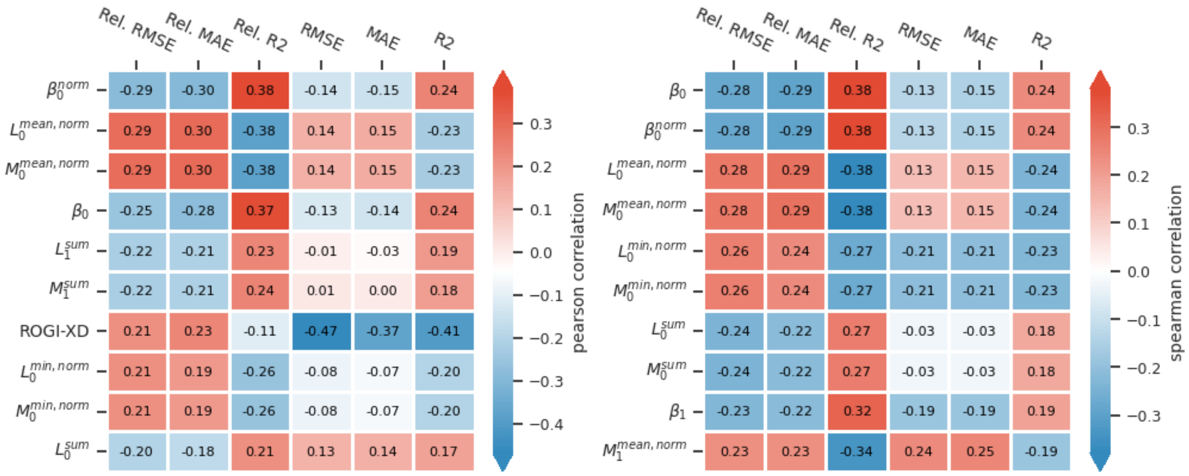

Figure S23: *Pearson and Spearman correlation between different topological metrics and test errors across all descriptor representations.*

SHAP summaries

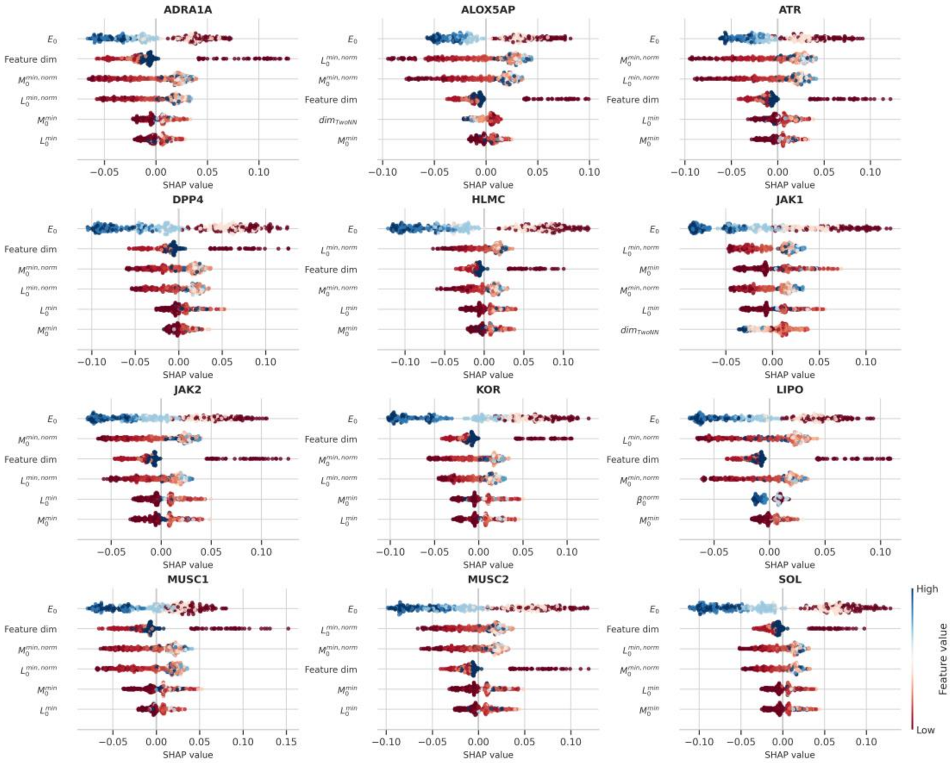

Figure S24: *SHAP summary for leave-one-dataset-out cross-validation models on randomly split data and embedding representations.*

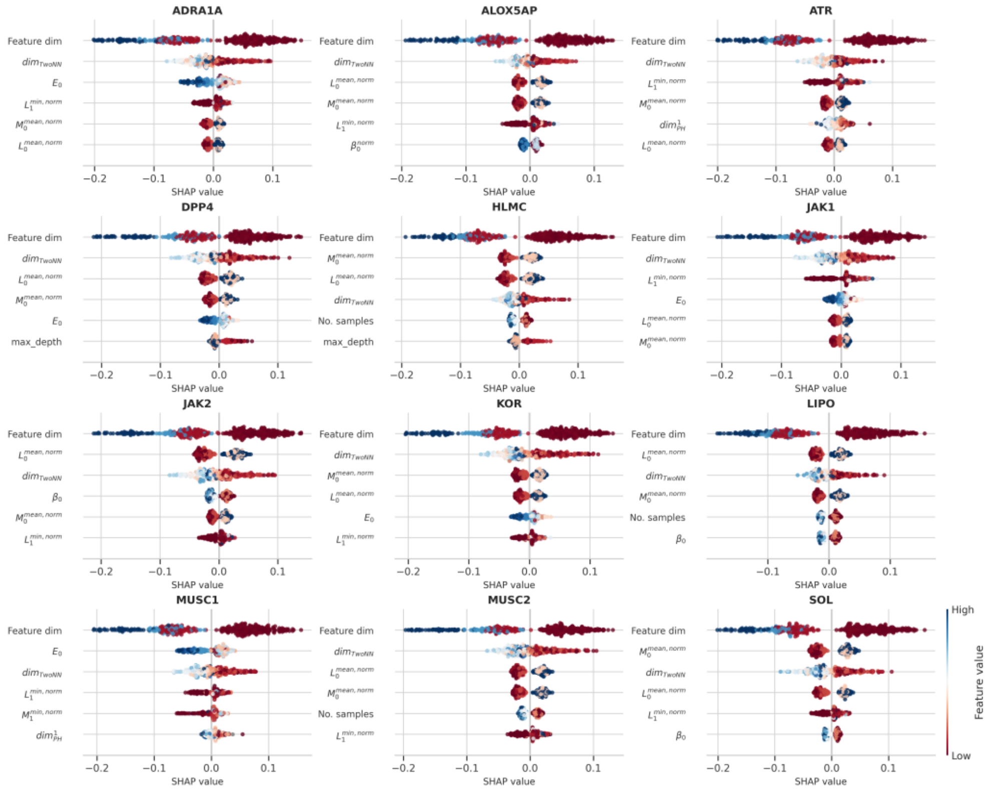

Figure S25: *SHAP summary for leave-one-dataset-out cross-validation models on randomly split data and fingerprint representations.*

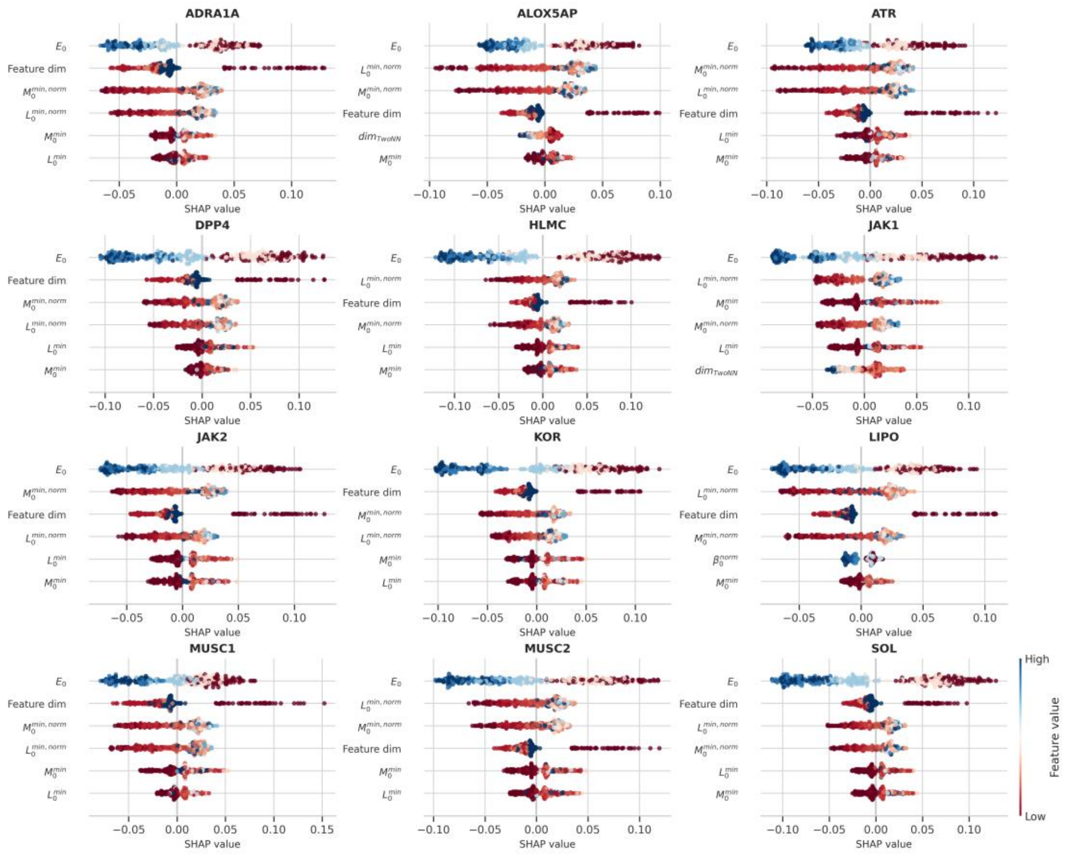

Figure S26: *SHAP summary for leave-one-dataset-out cross-validation models on randomly split data and descriptor representations.*

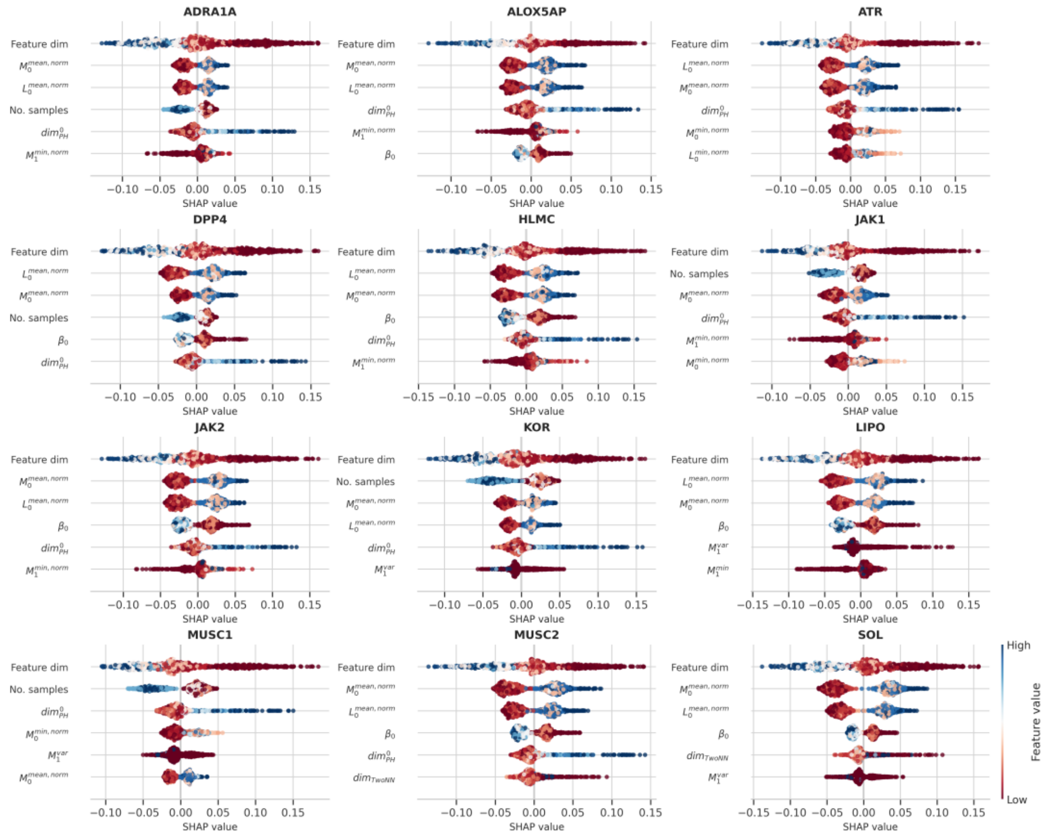

Figure S27: *SHAP summary for leave-one-dataset-out cross-validation models on scaffold split data and all representations.*

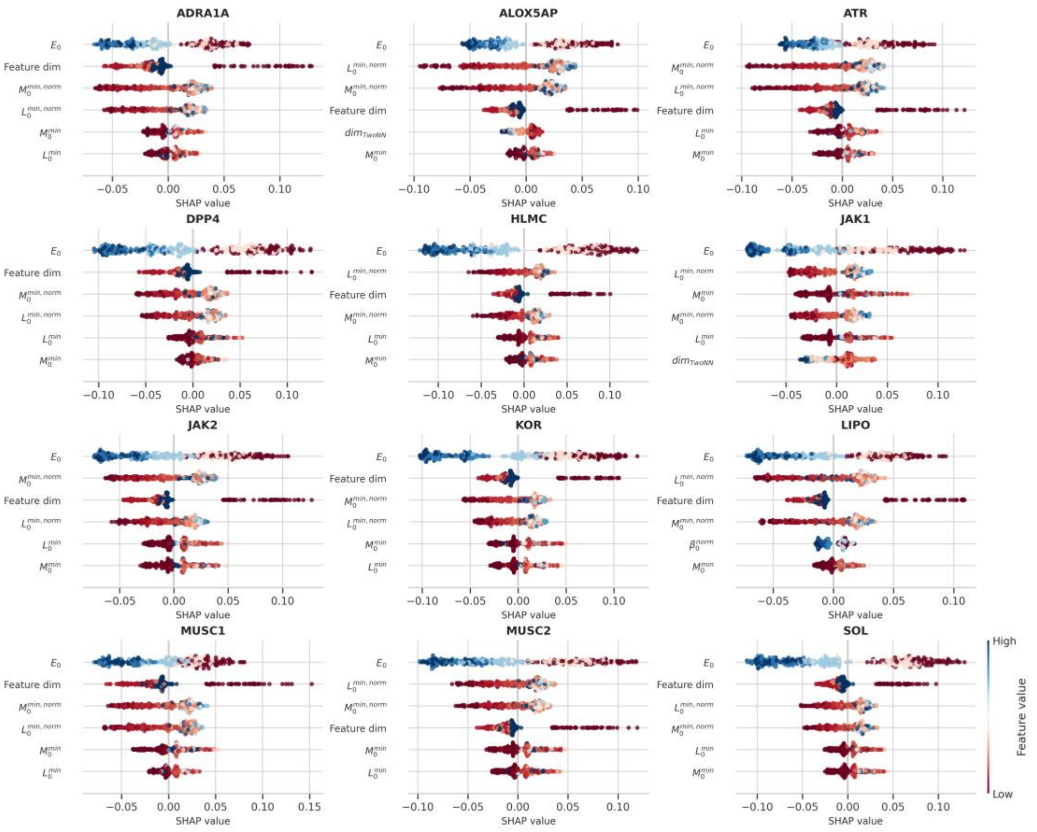

Figure S28: *SHAP summary for leave-one-dataset-out cross-validation models on scaffold split data and embedding representations.*

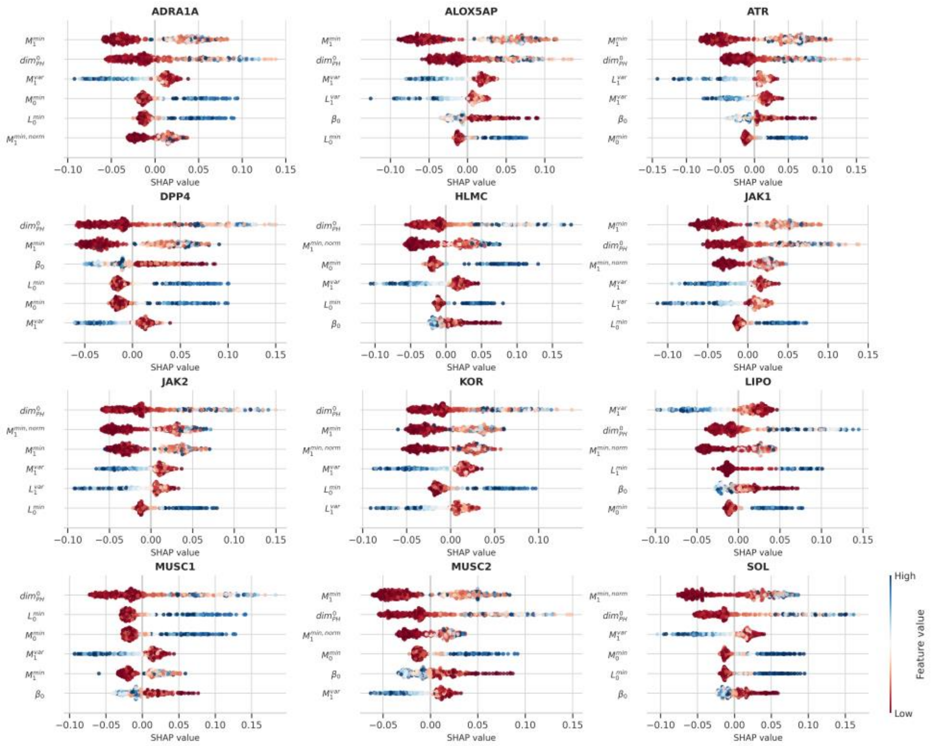

Figure S29: *SHAP summary for leave-one-dataset-out cross-validation models on scaffold split data and fingerprint representations.*

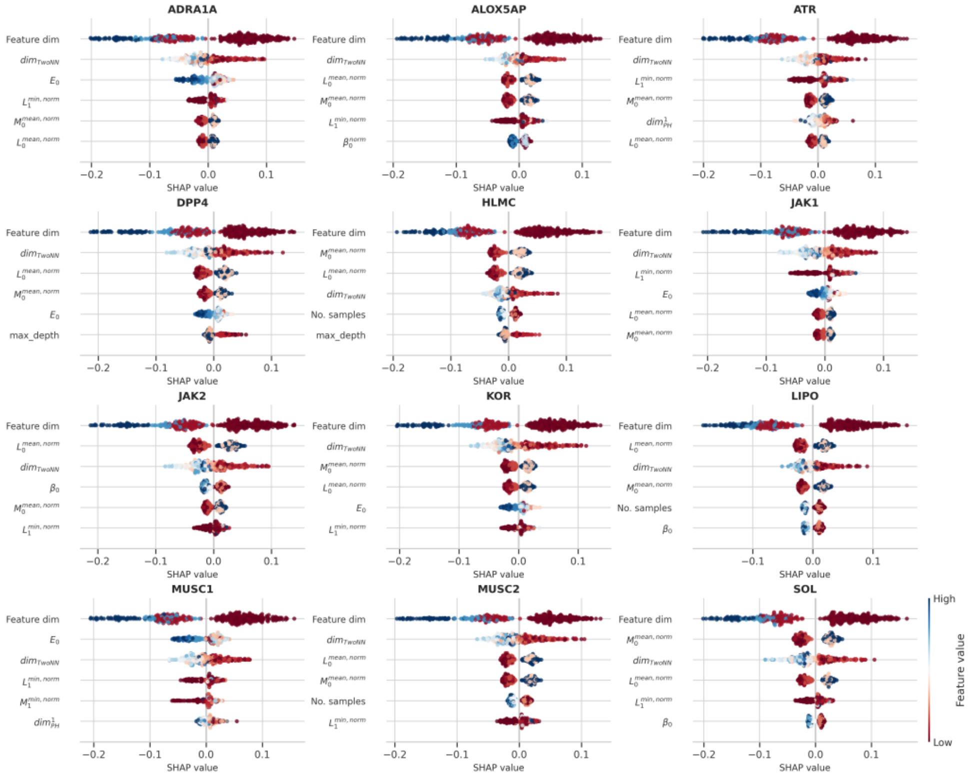

Figure S30: *SHAP summary for leave-one-dataset-out cross-validation models on scaffold split data and fingerprint representations.*
